# Defining an additivity framework for mixture research in inducible whole-cell biosensors

**DOI:** 10.1038/srep17200

**Published:** 2015-11-26

**Authors:** K. Martin-Betancor, C. Ritz, F. Fernández-Piñas, F. Leganés, I. Rodea-Palomares

**Affiliations:** 1 Departament of Biology, Facultad de Ciencias, Universidad Autónoma de Madrid, 28049 Madrid, Spain; 2 Department of Nutrition, Exercise and Sports, Faculty of Science, University of Copenhagen, Rolighedsvej 30, DK-1958 Frederiksberg C, Denmark

## Abstract

A novel additivity framework for mixture effect modelling in the context of whole cell inducible biosensors has been mathematically developed and implemented in R. The proposed method is a multivariate extension of the *effective dose* (*ED*_*p*_) concept. Specifically, the extension accounts for differential maximal effects among analytes and response inhibition beyond the maximum permissive concentrations. This allows a multivariate extension of *Loewe additivity*, enabling direct application in a biphasic dose-response framework. The proposed additivity definition was validated, and its applicability illustrated by studying the response of the cyanobacterial biosensor *Synechococcus elongatus* PCC 7942 pBG2120 to binary mixtures of Zn, Cu, Cd, Ag, Co and Hg. The novel method allowed by the first time to model complete dose-response profiles of an inducible whole cell biosensor to mixtures. In addition, the approach also allowed identification and quantification of departures from additivity (interactions) among analytes. The biosensor was found to respond in a near additive way to heavy metal mixtures except when Hg, Co and Ag were present, in which case strong interactions occurred. The method is a useful contribution for the whole cell biosensors discipline and related areas allowing to perform appropriate assessment of mixture effects in non-monotonic dose-response frameworks

The prediction of the expected effect of chemical mixtures when only the effect of individual components is known is a hot topic in pharmacology, toxicology, and ecotoxicology^1^,^2^,^3^. A central element in mixture research is the definition and mathematical formulation of additivity^2^,^4^,^5^. At present, there are two sound pharmacological definitions of additivity: *Loewe additivity*^6^ and *Bliss independence*^7^, which are the foundations of the so-called *Concentration addition* (CA) and *Independent Action* (IA) additivity models, respectively^8^. Departures from *Loewe additivity* can be quantitatively studied based on the *Combination Index* (CI)^4^,^9^. The crucial prerequisite for the applicability of any additivity model is to fulfill certain mathematical assumptions^5^. The basic mathematical feature of *Loewe additivity* is that the effects of the mixture components could be formulated in terms of a common *effective dose* (*ED*_*p*_). For instance, this requisite is trivially met when all individual mixture components present identical maximal effects, (see Fig. 1a). An important complication occurs with mixtures of chemicals that show differential maximal effects: When this happens, additivity hypothesis can only be formulated up to the effect levels achieved by the less potent compound present in the mixture due to inherent mathematical features of the additivity definition^5^ (Fig. 1b). An additional challenge for formulating the additivity hypothesis occurs when the studied response is susceptible to result in non-monotonic biphasic dose-response patterns (see Fig. 1c). Besides, in biphasic dose-response curves, two different concentrations can result in the same *fractional effect* (Fig. 1c), resulting in misleading conclusions^5^. These problems have hampered the applicability of additivity models in important areas where differential maximal effects and biphasic dose-response patterns are commonly observed, such as in hormetic effects^10^, hormone agonists/antagonists research^11^, AhR agonists/antagonists activity research^5^, endocrine disrupters activity research^12^,^13^, and in general in the field of inducible (turn-on) whole cell biosensors.

To overcome these bottlenecks, some authors recently suggested the need for a formal mathematical expansion of the additivity formulations that may allow working with differential maximal effects^14^,^15^. Other authors have proposed a pragmatic numerical approximation based on a toxic unit extrapolation method to solve the problem^5^.

Inducible whole cell biosensors are a paradigmatic case of a biological system displaying differential maximal effects and usually biphasic dose-response profiles (e.g.,^16^) (Fig. 1c). Whole cell biosensors are intact, living cells genetically engineered to produce a dose-dependent measurable signal in response to a specific chemical or physical stimulus in their environment^17^. Inducible whole cell biosensors response is usually characterized by a dose-dependent biphasic profile presenting an induction region up to a concentration threshold (*maximum permissive concentration*), and a subsequent inhibition region where the biosensor response decays, possibly due to the inherent toxicity of the analyte above certain concentrations which affects cell viability^18^. Whole cell biosensors have been extensively used in the last 3 decades for the detection and quantification of different analytes and stresses of interest (for a review see^17^,^19^). Despite they have a clear vocation to be applicable in realistic conditions, mixture effect research using inducible whole-cell biosensors is presently a poorly developed research area. From our point of view, the main reason is the lack of a founded theoretical basis and experimental additivity framework.

In the present work, a novel additivity framework for mixture research in the context of whole cell inducible biosensors has been mathematically developed. The method proposes a multivariate extension of the *effective dose* (*EDp*) to take into account the occurrence of differential maximal effects and inhibition beyond the MPCs. In effect, this allows an extension of *Loewe additivity* that enables its direct application in a biphasic dose-response framework. A family of user friendly utilities has been incorporated in the (*drc*) package^20^ for R. The method has been illustrated studying the response of the cyanobacterial biosensor *Synechoccocus elongatus* PCC 7942 pBG2120 to binary mixtures of 6 heavy metals (Zn, Cu, Cd, Ag, Co and Hg). *Synechococcus* sp. PCC 7942 pBG2120 bears a fusion of the promoter region of the *smt* locus of *Synechococcus* sp. PCC 7942 to the *luxCDABE* operon of *Photorhabdus luminescens*. It is an inducible self-luminescent *whole cell* biosensor able to respond to a broad range of heavy metal cations which present differential maximal effects and biphasic dose-response curves^16^. The method is a useful contribution for the entire whole-cell biosensors discipline and related areas which allows to perform sound mixture-effect research in the framework of biphasic dose-response curves.

## Theory

### A novel framework for mixture-effect research for whole cell biosensors

We propose a novel framework for modelling mixture effects in *whole cell biosensors* showing biphasic dose-response curves. It is characterized by the following 5 steps: (1) Fitting biphasic dose-response profiles. (2) A dimensional extension of the *effective dose* notation. (3) *Two-dimensional formulation of Loewe additivity*. (4) Prediction of mixture effects based on individual component biphasic dose-response data. (5) Analysis of departures from additivity.

### Fitting biphasic dose-response profiles

**Figure 1:**
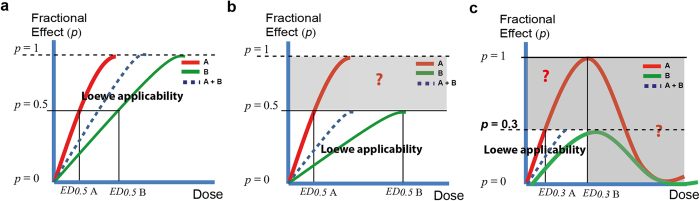
Applicability of Loewe additivity. Typical dose-response profiles for (**a**) classical monotonic dose-response curves for chemicals A and B showing identical maximal effects, (**b**) classical monotonic dose-response curves for chemicals A and B presenting differential maximal effects, and (**c**) biosensor type biphasic dose-response curves for chemicals A and B presenting differential maximal effects and toxicity threshold. The meanings of the terms presented in the figure can be found in theory section.

Inducible whole cell biosensors usually present inverted *v-shaped* biphasic dose-response profiles (Fig. 1c). This specific type of dose-response pattern may be fitted using nonlinear regression model equations Gaussian and LogGaussian. We considered 2 specific inverted *v-shaped* functions *f*: the *Gaussian* (Eq. 1 below) and the *log Gaussian* (Eq. 2 below) equations, which are defined as follows:


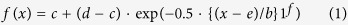



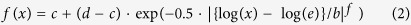


where the parameters c and d correspond to the limits for x = 0 and x tending to infinity and the parameters b and e control the steepness of the curve and location of the peak, respectively. The parameter f describes asymmetry in the curve (i.e., asymmetry between the left and right sides of the peak).

### A multivariate extension of the effective dose notation

**Figure 2:**
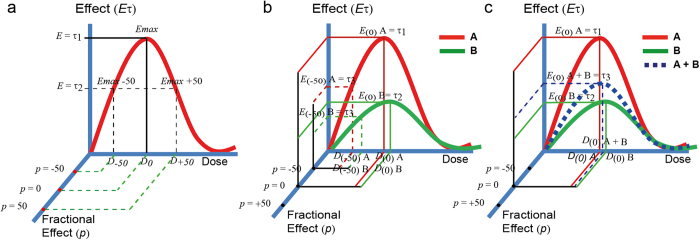
The bi-dimensional fractional effect notation. (**a**) Fractional *effective doses ED*_*p*_ in biphasic dose-response systems is proposed to be scaled as fractions (*p*) of the *E*_*max*_ defining the fractional effect scale (*Ep*). The empirical effect scale (*E*_(τ)_, in the *y* axis) is decoupled from the fractional effect scale (*Ep*) which is projected in the *z* plane. This allows to define fractional effects covering the entire biphasic dose-response curve. (**b**) The proposed *fractional effective* notation allows to scale in a common fractional effect scale *stimuli* A and B showing differential maximal effects. (**c**) An additive biphasic dose response pattern “A + B” can be formulated for a theoretical mixture of A and B based on the univocal relationship of the fractional effect scale (*Ep*) with the other two dimensions: *D*_*(p)*_ and *E*_*(p)*_. For notation meanings in the Figures see section 2.

In the present work, a notation for fractional *effective doses ED*_*p*_ scaled from the MPCs (hereafter, *E*_*max*_) has been chosen (Fig. 2a). The novelty of the approach is that we maintain the empirical effect scale (*E*_(τ)_) in the *y* axis, and we project the fractional effect scale (*Ep*_*(p)*_) in the *z* plane (see Fig. 2a). Fractional effects (*p*) in the *Ep* scale are defined as follows: −100 ≤ *p* ≤ 100. For *p* < 0, fractional effects on the left side of the *E*_*max*_ are obtained (Fig. 2a) (the induction part of the curve), for *p* > 0, fractional effects on the right side of the *E*_*max*_ are obtained (the inhibition part of the dose-response curve). *p* = 0 = *E*_*max*_ = MPC. Decoupling the fractional effect scale (*Ep*_*(p)*_) from the empirical effect (*E*_(τ)_) allows to scale inverted *v-shaped* dose-response curves with differential maximum effects in a unique fractional scale independently of the *maximum* level of effect (*E*_*max*_) attained (Fig. 2b). However, *ED*_*p*_ needs to be dimensionally extended to account for differences in both the dose (*D*) and effect (*E*) scales (Fig. 2b). Therefore we define *ED*_*p*_ as a two-dimensional vector (*D*_*(p)*_, *E*_*(p)*_) where *D*_*(p)*_is the dose required to get the desired fractional effect (*p*) (i.e. 50%), and *E*_*(p)*_ is the effect in the empirical effect scale (*E* (τ)) achieved at this fractional effect (*p*).

### Two-dimensional formulation of Loewe additivity

For classical monotonic dose-response curves, a uni-dimensional effective dose-notation and additivity formulation are enough to perform accurate additive predictions. In the same way that the fractional notation (*ED*_*p*_) needs to be extended to set a proper common fractional effect scale in biphasic dose-response curves, the existing additivity formulation needs to be extended as well. For this we propose a two-dimensional formulation of *Loewe additivity* computed for the two components of *ED*_*p*_ = (*D*_*(p)*_, *E*_*(p)*_)_*p*_ (Fig. 2c). The additivity formulation on the dose (*D*) scale is identical to the original *Loewe additivity* formulation. For notational convenience, we will formulate hereafter *Loewe additivity* as follows:


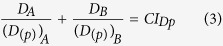


where *D*_*A*_ and *D*_*B*_ are the doses of the components A and B which in combination produces a fractional effect *p* on the measured biological response. *(D*_*(p)*_)_*A*_ and *(D*_*(p)*_)_*B*_ are the doses that individually result in a fractional effect *p* for the components A and B, respectively. In case (Eq. 3) equals 1 *Loewe additivity* holds. Otherwise, Combination Index Theorem holds^4^. If 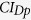 denotes the Combination Index in the *D* dimension (see Fig. 2c) for the considered fractional effect *p*^4^, 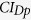 < 1 indicates synergism, 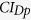 > 1 indicates antagonism. To project *Loewe additivity* to the empirical effect dimension (*E(*τ)), we simply assume that the effect (*E*) dimension is equivalent to the Dose (*D*) dimension in its relationship with the fractional effect (*Ep*) dimension (Fig. 2c). Therefore, it holds that *Loewe additivity* is computed in the *E* dimension as follows:


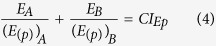


where *E*_*A*_ and *E*_*B*_ are the effects (in the empirical scale (τ)) of the components A and B which in combination results in a *fractional effect p* on the measured biological response. (*E*_*(p)*_)_*A*_ and (*E*_*(p)*_)_*B*_ are the *effects* in the empirical effect scale of the components A and B resulting individually in the desired *fractional effect p*. 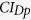, is the Combination Index in the *E* dimension for the considered fractional effect *p*.

The proposed two-dimensional formulations are susceptible of extension to *n* components as follows:


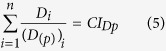



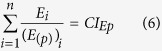


where *D*_*i*_is the dose of the *i*^*th*^ component which in combination produces a fractional effect *p*. *(D*_*(p)*_)_*i*_ is the dose of the *i*^*th*^ components that individually produces the same fractional effect *p*. *E*_*i*_is the effect (τ) of the *i*^*th*^ component which in combination produces a fractional effect *p*. *(E*_*(p)*_)_*i*_ is the effect (τ) of the *i*^*th*^ components that individually produce the same fractional effect *p*. Thus, *CI*_*Dp*_and *CI*_*Ep*_ are the combination index scores for the *D* and *E* dimensions at any fractional effect *p*.

### Predictions of the joint effect of a mixture under two-dimensional Loewe additivity

Equation (5) for CI_*Dp*_ = 1 can be rewritten in a predictive formulation (allowing for *in-silico* predictions of the joint effect of *n* mixture components based on individual chemical information only) according to Faust, *et al*.^21^, if *D*_*i*_ are expressed as relative proportions *j*_*i*_ of the total dose (*D*_*mix*_), where *j*_*i*_ = *D*_*i*_/*D*_*mix*_. Under the condition that the mixture elicits a *p* total fractional effect, the total dose of the mixture *D*_*mix*_ is defined as the effective dose of the mixture (*D*_*pmix*_) required to produce a fractional effects *p*. If CI = 1, ⇒*D*_*i*_ can be substituted in Eq. (5) by *j*_*i*_ *D*_*p mix*_, and by rearrangement, it holds:


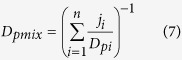


According to Gonzalez-Pleiter, *et al*.^22^, equation (5) can be similarly rewritten as follows when CI ≠ 1:


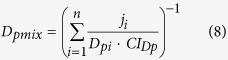


Considering the proposed two-dimensional notation *ED*_*p*_ = (*D*_*(p)*_, *D*_*(p)*_), the formulation of Equation (7) in the *E* dimension gives:


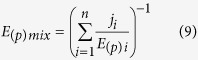


In case CI ≠ 1:


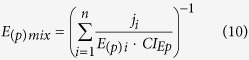


### Analysis of departures from additivity

The definition in equations (5) and (6) also allows investigating departures from additivity in biphasic dose-response systems. The result from evaluating equations (5) and (6) are two numerical values of the Combination Index (*CI*) for the *D* and *E* dimensions, which can be expressed as a two-dimensional vector *CI*_*p(D, E*)_ = (CI_*D p*_, CI_*E p*_). This approach yields 9 theoretical combinations of CI_*p* (*D,E*)_ as follows: CI_*p* (*D,E*)_ = (1, 1); (1, <1); (1, >1); (<1, 1); (<1, <1); (<1, >1); (>1, >1); (>1, 1); (>1, <1).

For global assessment of departures from additivity, a simplification of the two-dimensional information can be obtained by calculating a weighted index CI_*wp*_ for any fractional effect *p* as follows:


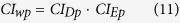


where CI_*wp*_ > 1 indicates overall antagonism, CI_*wp*_ = 1 an overall additive effect, and CI_*wp*_ <1, an overall synergistic effect.

## Results

**Figure 3:**
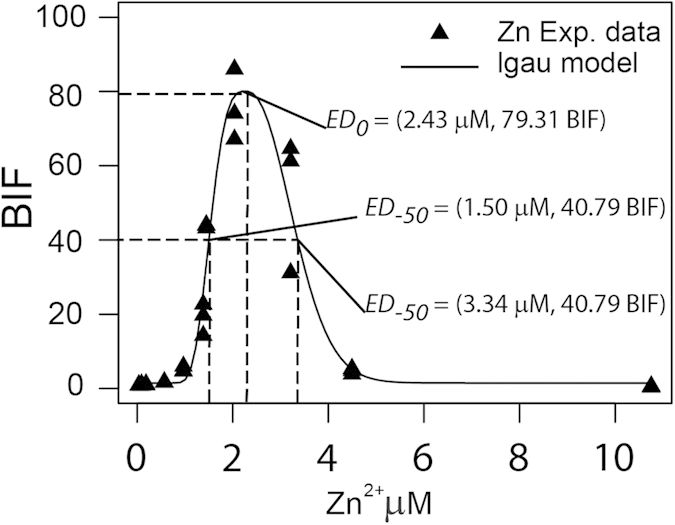
Fitting biphasic profiles with non-linear regression models. Experimental response of *Synechoccocus elongatus* PCC 7942 pBG2120 to increasing concentrations of Zn^+2^. Best fit model for Zn^+2^ logGaussian “lgau2” (see Supplementary Material SM 1) is presented in the figure. Position of selected *ED*_*p*_ vectors (−50, 0, + 50) are marked in the figure.

Dose-response profiles of *Synechoccocus elongatus* PCC 7942 pBG2120 exposed to Zn, Cu, Cd, Ag, Co and Hg and their binary combinations were fitted using the proposed set of *v-shaped* non-linear functions described in Theory section. Best fit models for each metal and metal mixture were selected accordingly to the minimum of the residual sum of squares^20^. Supplementary Material SM1 shows a summary of the selected model function and parameters for each individual metal and metal mixture. Overall, the majority of single metal experimental data were better fitted by log Gaussian function, and only Ag was fitted better by a Gaussian model. On the contrary, the responses of the biosensor to half of the binary metal mixtures were fitted better by Gaussian, and the other half by logGaussian model (see Supplementary Material SM1). Figure 3 shows as an example, the goodness of fit of the *v-shaped* non-linear function logGaussian “lgau2” to Zn response experimental data (see Supplementary Material SM1 for model parameters). As can be observed, the function fits adequately Zn experimental data at both the induction and the inhibition part of the dose-effect curve. Using the *drc* package we can get *ED*_*p*_ vectors (*D*_*(p)*_*, E*_*(p)*_) at any desired fractional effect *p*. For example, we can calculate the Zn *ED*_*−50*_ = (1.50 μM, 40.79 BIF), Zn *ED*_*0*_ = (2.43 μM, 79.31 BIF) and the Zn *ED*_*+50*_ to be (3. 34 μM, 40.79 BIF) (Fig. 3) (definitions in Methods section). Since *ED*_*p*_ is two-dimensional, it includes not only the concentration required to produce a specific fractional effect *p*, but also the actual BIF that the biosensor signal achieves at this specific fractional effect for a specific analyte. A summary of *ED*_*p*_ vectors (−50, 0, +50) for the 6 metals can be found in Supplementary Material SM2. As can be seen, the bi-dimensional notation allows to explicitly predict concentrations of the analyte resulting in fractional effects in both the induction and the inhibition regions of the dose-response curves. An illustrative example on how to get *ED*_*p*_ vectors can be found in Supplementary Material SM3.

**Figure 4:**
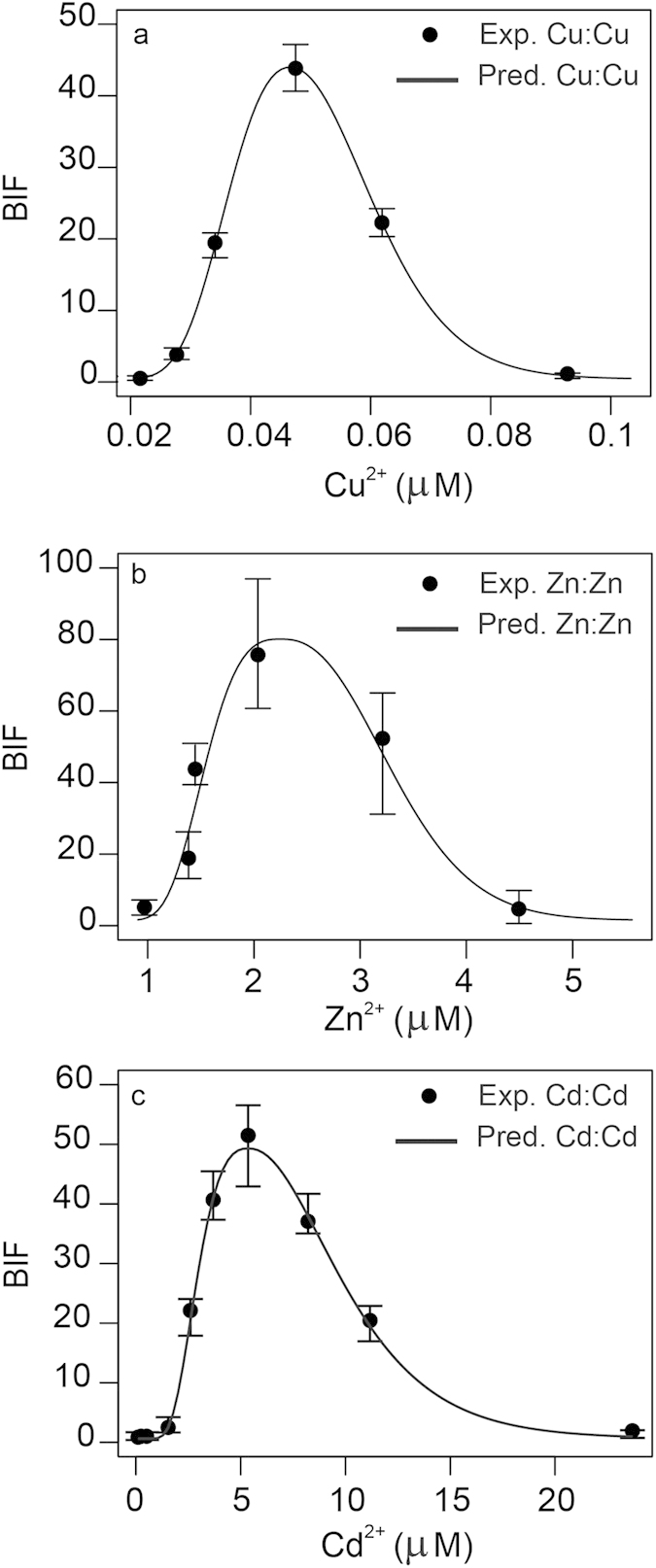
Validation of the additivity definition using sham mixtures. Predicted dose-response patterns of sham binary mixtures for (**a**) Cu:Cu, (**b**) Zn:Zn, (**c**) Cd:Cd. Predicted dose-response patterns were calculated solving the multivariate extension of *Loewe additivity* equation in predictive formulation (eqs (7) and (9)). Error bars are standard errors (*n* = 3–4).

Once individual metal biphasic response profiles are adequately fitted, and *ED*_*p*_ vectors can be obtained to any fractional effect *p*, it is possible to predict the biosensor response to any combination of the individual metals evaluating Equations (7) and (9). However, prior to any further analysis, it is required to validate the proposed two-dimensional definition of *Loewe additivity*. Figure 4 shows predicted *vs* experimental dose-response patterns of sham binary mixtures for Zn:Zn, Cd:Cd and Cu:Cu. A near perfect overlap occurred between the experimental and predicted dose-response curves of sham mixtures for the 3 metals. This validates the proposed multivariative formulation of *Loewe additivity* model for biphasic dose-response curves, even at mixture concentrations where inhibition of the signal occurs (See Fig. 1b).

**Figure 5:**
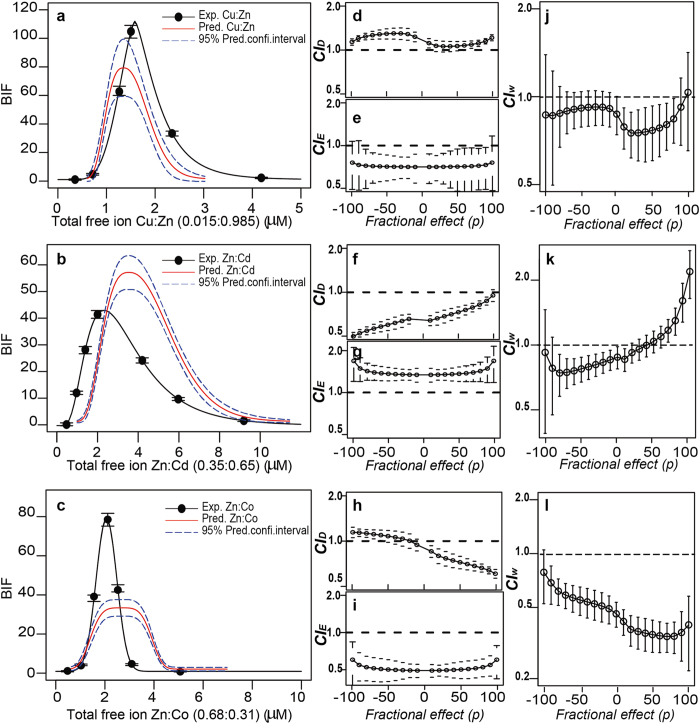
Analysis of departures from additivity. Experimental *vs* predicted (under additivity) dose-response patterns for Cu:Zn (**a**), Zn:Cd (**b**) and Zn:Co (**c**) binary mixture´s, respectively. Extended *p-CI* plots presenting departures from additivity (as CI values) as a function of the effect level (*p*) for the *D* dimension of Cu:Zn (**d**), Zn:Cd (**f**) and Zn:Co (**h**). Extended *p-CI* plots presenting departures from additivity (as CI values) as a function of the effect level (*p*) for the *E* dimension of Cu:Zn (**e**), Zn:Cd (**g**) and Zn:Co (**i**). Extended *p-CI*_*w*_ plots presenting weighted departures from additivity (as CI_*w*_ values) as a function of the effect level (*p*) for Cu:Zn (**j**), Zn:Cd (**k**) and Zn:Co (**l**). Error bars are standard errors (*n* = 3–4). Total free ion concentrations presented in the Figures are those of Supplementary Material SM2 presented as *ED*_*0*_ in Supplementary Material SM2 corrected by MINTEQ calculations due to the presence of the two metals used in each mixture.

Once the multivariative extension of *Loewe additiv*ity was validated with the sham mixtures, we applied the method to perform additivity predictions and to study the nature of the interaction (if any) of the response of *Synechoccocus elongatus* PCC 7942 pBG2120 to the 15 possible binary mixtures of Zn, Cd, Cu, Ag, Hg and Co. Complete results of the analysis can be found in Supplementary Material SM5. A representative selection of the results is presented in Fig. 5. In the case of the binary mixture Cu:Zn (Fig. 5a), the additivity prediction fitted reasonably well the experimental *dose-response* profile. However, the additivity predictions of the binary mixtures Zn:Cd (Fig. 5b) and Zn:Co (Fig. 5c), deviated from their respective experimental dose-response pattern, suggesting a departure from additivity. However, which kind of departure are we observing?

To answer this question, a quantitative analysis of the nature of the interactions is required^3^,^4^. Departures from additivity can be quantified solving Equations 5 and 6 for any fractional effect *p*. As illustrative example, we used *extended p-CI* plots to graphically present the results of the quantification of the interactions of the three selected mixtures (Cu:Zn, Zn:Cd and Zn:Co). *Extended p-CI* plots allow to observe the nature of the interactions along the *D* and *E* dimensions covering the complete biphasic dose-response curves, representing the deviation from the additivity line (CI = 1) for any fractional effect *p*. Figures 5d,f,h and Figure 5e,g,i show extended *p-CI plots* for the *D* and *E* dimensions (respectively), for the three selected metal binary mixtures (Cu:Zn, Zn:Cd and Zn:Co). *Extended p-CI plots* for *D* and *E* axis (Fig. 5d,e, respectively) for the Cu:Zn combination showed CI values near 1 (the additivity line) along the entire range of fractional effects (*p*) in both dimensions, confirming the near additive behaviour of this metal combination. The Zn:Cd binary combination presented statistically significant synergism (CI <1, *p* < 0.05) along the entire range of fractional effects (*p*) in the *D* dimension (Fig. 5f), but statistically significant antagonism (CI >1, *p* < 0.05) in the *E* dimension (Fig. 5g). On the other hand, the Zn:Co combination showed a *p*-dependent interaction patters in the dose (*D*) axis (going from antagonistic to synergistic), and consistent synergism (CI <1, *p* < 0.05) in the effect (*E*) axis along the entire range of effect levels (*p*) (Fig. 5h,i). See Supplementary Material SM4 for specific *p*-values for three selected fractional effect levels (−50, 0, +50). Figure 5j–l showed *Extended p-CI*_*w*_ *plots* for the mixtures Cu:Zn, Zn:Cd and Zn:Co, respectively. *Extended p-CI*_*w*_ *plots* (see equation 11) can be used as a measure of the overall fitness to additivity of the response of a biosensor to mixtures of analytes. As can be seen in the Fig. 5j, the overall effect of the combination of Cu:Zn combination was additive, that of Zn:Cd combination was *p*-dependent (Fig. 5k), going from synergistic (below the MPC) to antagonistic (above the MPC). That of Zn:Co was consistently synergistic CI < 0.5 along the entire range of effect levels (*p*) (Fig. 5l).

**Figure 6:**
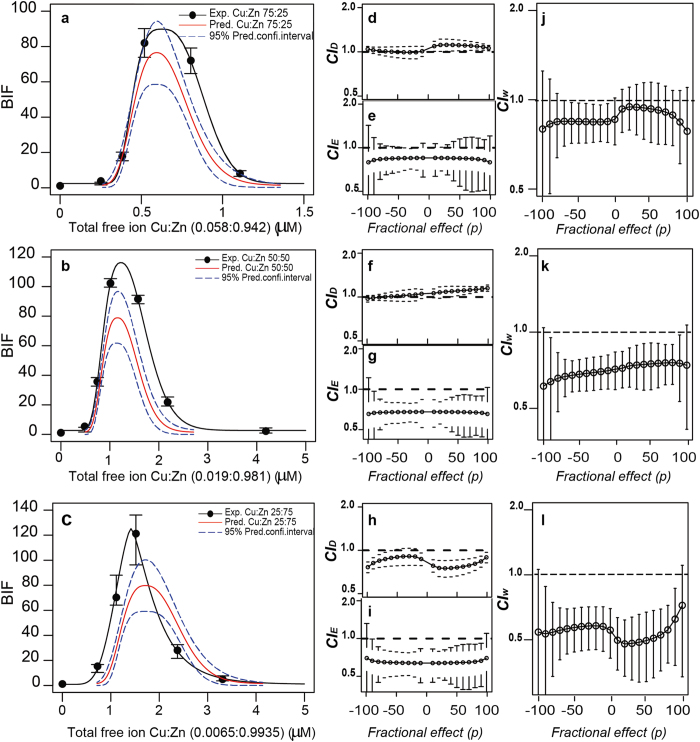
Effect of mixture ratio. Experimental *vs* predicted (under additivity) dose-response patterns for the mixture Cu:Zn at 75:25 (**a**), 50:50 (**b**) and 25:75 (**c**) ratios based on the *D*_*0*_ concentration of the metals. Extended *p-CI* plots presenting departures from additivity (as CI values) as a function of the effect level (*p*) for the *D* dimension (**d**,**f**,**h**), and the *E* dimension (**e**,**g**,**i**). *Extended p-CI*_*w*_ *plots* presenting weighted departures from additivity (as *CI*_*w*_ values) as a function of the effect level (*p*) for the binary mixture Cu:Zn at 75:25 (**j**), 50:50 (**k**), 25:75 (**l**) mixture ratios. Error bars are standard errors (*n* = 3–4). Total free ion concentrations presented in the Figures are those presented as *ED*_*0*_ in Supplementary Material SM2 corrected by MINTEQ calculations due to the presence of the two metals used in each mixture.

The effect of the metal ratio on the predictive power of the multivariative extension of Loewe additivity was addressed for selected binary mixtures. Figure 6a–c shows the experimental dose-response patterns and the respective additivity predictions for the three different metal ratios (75:25, 50:50, 25:75, respectively) of the binary metal mixture Cu:Zn. *Extended p-CI plots* for the D and E dimensions for the different metal ratios are presented in Fig. 6d–i. In addition, *extended p-CI*_*w*_ *plots* are presented in Fig. 6j–l. The selection of metal ratios allows to specifically address the possibility of predicting the dose response patterns including metal combinations in which eventually one of the metal may be present below the MPC and the other above the MPC and vice versa. As can be seen in Fig. 6, the main features of the dose-response pattern of the 50:50 mixture of Cu:Zn (Fig. 6b), that is additivity in *D*, synergism in *E*, and overall additive effect (CI_*w*_ > 0.5) is essentially conserved in the 25:75 and the 75:25 ratio. The only differences were the occurrence of a slight tendency to synergism in *D* in the 25:75 ratio (Fig. 6h), and a slight tendency to synergism in both the 75:25 and 25:75 ratios based on CI_*w*_ (but still additive based on the management criterion: 0.5 < CI_*w*_ < 2). Similar results were obtained for ratio variations for the mixture Zn:Cd (Supplementary Material SM4).

**Figure 7:**
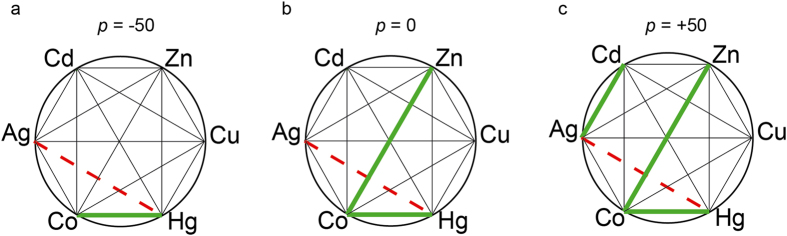
CIw Polygonograms. Polygonograms summarizing weighted combination index (CI_*w*_) for all the binary combinations of the 6 studied metal cations (Zn, Cu, Cd, Ag, Co and Hg) at three representative fractional effects (*p*): −50 (**a**), 0 (**b**), +50 (**c**). Departures from additivity are based on the risk management criterium (see Methods). Thin solid black lines represent additive effect (0.5 < CI_*w*_ < 2). Solid green lines indicate synergism (CI_*w*_ < 0.5). Dashed red lines indicate antagonism (CI_*w*_ > 2).

The analysis of the departures from additivity for the 15 possible binary combinations of the studied metals revealed a complex scenario where the 9 possible theoretical combinations of the CI_*D,E*_ vectors anticipated in Theory section were actually found (Supplementary Material SM5). In order to get a global idea on the fitness to additivity of the response of the biosensor to the binary mixtures of metals, we computed CI_*w*_ values according to Equation (11) which were summarized as well as *polygonograms*^4^ (for *p* levels −50, 0, +50) in Fig. 7. Interestingly, additive or near additive effects (according to the management criterion: 0.5 < CI_*w*_ < 2) hold for binary mixtures of Zn, Cd, Ag and Cu at the three representative *p* levels (Fig. 7). However, some mixtures containing Hg, Co and Ag resulted in significant departures from additivity: The mixtures Hg:Co and Hg:Ag resulted in synergism and antagonism, respectively at the three representative *p* levels (−50, 0, +50). In addition, some mixtures resulted in effect-level dependent departures from additivity: the mixture Co:Zn resulted in synergism at *p* = 0 and *p* = +50, and the mixture Ag:Cd resulted in synergism at *p* = +50.

## Discussion

Here we present a theoretical framework which allows to perform sound mixture-effect research in inducible whole cell biosensors and related fields. To have a mathematical formulation of additivity is crucial in order to obtain accurate and comprehensive results in mixture research as demonstrated in the last 20 years in pharmacology and eco/toxicology^2^,^3^,^4^. In these disciplines, *Loewe additivity* is the gold standard for additivity formulations^2^,^3^,^4^. However, the practical applicability of *Loewe additivity* is historically hampered in biological systems presenting differential maximal effects and non-monotonic responses such as biphasic dose-response curves^5^,^14^. In the present work we propose a multivaritive extension of *Loewe additivity* which allows its application in the context of differential maximal effects and biphasic dose-response curves. The proposed methodology was validated and tested using the inducible whole-cell self-luminescent metal biosensor *Synecchococus elongatus* PCC 7942 pBG2120 as case study. Our solution is in agreement with the conceptual formulations which Belz, *et al*.^14^ proposed in order to extend the applicability of *Loewe additivity* in the context of hormesis (an stimulatory effect found at low doses of toxicants). They postulated that differential maximal hormetic effects among individual mixture components force the need to perform additivity formulations in both dose (*D*) and effect (*E*) dimensions independently. As solution, they proposed to use the original *Loewe* equation (Equ. 3) for predictions in the (*D*) dimension, but a simple summation of fractional effects for predictions in the *E* dimension. Their predictions were reasonable for the dose (*D*) dimension, but were, in their own words “more dubious” for the (*E*) dimension^14^. This is because their proposal of *Loewe additivity* formulation for the *E* dimension was not accurate. We have demonstrated with the sham mixtures that our definition (Eqs 4 and 6) is a true projection of *Loewe additivity* in the (*E*) dimension. Scholze, *et al*.^5^ recently proposed an elegant approach to the differential maximal effect problem. Since, without multivariate extension, it is impossible to solve *Loewe additivity* equation for systems presenting differential maximal effects, they proposed a numerical approximation as a solution. Basically, they constructed an extrapolated interval of mixture predicted effects based on reasonable maximal and minimal hypothetical contributions of the less potent mixture components. The method worked well with a biosensor based on partial agonists of aryl hydrocarbon receptor (AhR) used for mixtures including components with varying maximal effects. However, they recognized that it cannot work in systems presenting inhibitory thresholds beyond the MPCs^5^.

The availability of a *Loewe additivity* formulation conceived for inducible whole-cell biosensors is an important milestone in this field of research which may allow for a wider generalization of mixture-effect research. This is especially true if a user friendly utility is available. We have made available all the presented mathematical equations, statistical and graphical utilities in the “dose-response curve” (*drc*) package for R^20^. Currently the methodology is presently set up for binary mixtures, and only two biphasic models (Gaussian and logGaussian) are available. However, future work will focus on extending the framework to *n*-component mixtures and more biphasic models.

An important application of the methodology is the possibility, by the first time, of predicting inducible whole cell biosensor responses to mixtures using single chemical experimental information only. As shown by the experiments with varying mixture ratios, this was true even when individual chemicals were present above or below the MPCs. This equals the potential applicability of inducible whole cell biosensors in mixture risk assessment with the same conditions and scope than those of monotonic toxicity tests, or *turn-off* whole cell biosensors such as Microtox^23^,^24^. This increases the opportunities of inducible whole cell biosensors to be included in future regulatory frameworks which will explicitly consider mixture risk assessment^1^,^25^. A complementary application of the methodology is the possibility of investigating departures from additivity (synergism and antagonism)^3^,^4^. In the presented case study with *Synechoccocus elongatus* PCC 7942 pBG2120, we found that the response to the binary combinations of the different metals was quite complex when analyzing the two constitutive dimensions (*D* and *E*), ranging from additivity to synergism and antagonism. In addition, we found that additivity, synergism or antagonism or any of their permutations, even of opposite effects occurred, for example a combination of synergism in (*D*), and antagonism in (*E*). In fact, the occurrence of opposite behaviours in the (*D*) and (*E*) dimensions was relatively common. One may wonder what the biological meaning of these patterns is. Most possibly, the answer will depend on the biological receptor used for the biosensor construction. In our case, the observed opposite behaviors in (*D*) and (*E*) dimensions for some metal mixtures may reflect the counteracting effects of the metals on the two independent but co-existing biological processes involved in the bioluminescence signal emission: On one hand, a synergistic effect of the metals on the induction of the *smtAB* promoter may result in lower metal concentration required to start to induce the system (synergism in *D*). However, a synergism in the toxicity of the metals affecting cell metabolism may result in a lower FMNH_2_ and ATP pool, inhibiting the bioluminescence signal (antagonism in *E*). Similar arguments may hold for the different possible combinations of departures from additivity in the two dimensions for other biosensor systems. However, from a practical point of view, one may be more interested in finding out whether or not an overall deviation from additivity in the biosensor response may likely occur with a specific combination of analytes, rather than in a detailed analysis of deviations in the *D* and *E* dimensions and their causes. In such case, CI_*w*_ = CI_*D*_ · CI_*E*_, can be used as a measure of the overall fitness to additivity of the response of a biosensor to mixtures of analytes. Intuitively, CI_*w*_ will tend to zero when CI_*D*_ and CI_*E*_ have opposite values, reflecting the counteracting effects of the tendencies in both dimentions. In the other hand, CI_*w*_ would be magnified (either in synergism or antagonism) when both CI_*D*_ and CI_*E*_ show the same kind of interaction. For example, if CI_*D*_ = 0.5 and CI_*E*_ = 0.5, CI_*w*_ = 0.25, this results in increased synergism in the overall response of the biosensor (lower concentration and higher induction than expected to reach the same *p* level). Therefore, CI_*w*_ will allow to easily detect those metal combinations which may result in evident deviations from additivity which may potentially be problematic from a practical view-point. The analysis of CI_*w*_ of the biosensor response for the 15 binary metal combinations revealed that the weighted response of *Synechoccocus elongatus* PCC 7942 pBG2120 could be considered nearly additive for the mixtures including Cu, Cd, Zn. However, important departures from additivity occurred when Hg, Co and to a lesser extent Ag were present in the mixtures. It is interesting to note that Hg and Co are the weakest inducers of the *smtAB* promoter alone^16^, however their impact on the analytical performance of the biosensor would be important. A similar analysis can be applied to any other biosensor strain whose robustness to interaction effects with detectable analytes would be addressed. Despite in the present work we have not performed any correction of the biosensor signal with a cell viability control which may serve to increase the dynamic range or bioanalytical performance of a whole-cell biosensor^17^,^18^, the presented analytical method (additivity formulations and analysis of departures from additivity) is equally applicable when the biosensor signal is corrected by such viability control. Finally, since our solution ultimately relies on a multivariate extension of the effective dose notation, the method may be generalized to other additivity formulations, such as *Bliss independence*^7^. *Bliss independence* may be a functional additivity formulation for stimuli with dissimilar mode of action but whose effects converge in the activation of a common biological effect^26^. Similarly, the bi-dimensional *Loewe additivity* may be generalized to any number of *k* extra dimensions which may be required to describe any biological response. For example, one important dimension, which may be approached with the same multivariative extension, is the exposure time. It may be done defining a three-dimensional *Loewe additivity* formulation, and an extension of the effective dose notation in the form *ED*_*p*_ = (D_*(p)*_, E_*(p)*_, t_*(p)*_). This method may have important applications in the formulation of additivity hypothesis in studies which may explicitly consider toxicokinetics and toxicodynamics, such as transcriptomics^27^.

## Conclusions

A multivariative extension of the *effective dose* (*ED*_*p*_) notation in order to take into account the occurrence of differential maximal effects and signal inhibition beyond the MPCs in biphasic dose-response curves has been developed. This allows a multivariative extension of *Loewe additivity* enabling its direct application in a biphasic dose-response framework. The proposed additivity definition has been experimentally validated using sham mixtures, finding excellent agreement between experimental and predicted dose-response patterns. The method was applied to study the response of the cyanobacterial self-luminescent metallothionein-based whole-cell biosensor *Synechoccocus elongatus* PCC 7942 pBG2120 to binary mixtures of 6 heavy metals (Zn, Cu, Cd, Ag, Co and Hg). The response of *Synechoccocus* to the mixtures can be considered nearly additive except when Hg, Co and to lesser extent Ag were present in the mixtures which resulted in important departures from additivity. The method has different applications and is a useful contribution for the entire whole-cell biosensors field and related areas allowing to perform sound mixture research in non-monotonic dose-response frameworks.

## Material and Methods

### Chemicals

Chemicals were of analytical grade, culture media and heavy metals stock solutions were prepared in MilliQ water. Heavy metal salts ZnCl_2_, CdCl_2_, AgSO_4_, CuSO_4_, HgCl_2_, CoCl_2_, PbNO_3_, MgCl_2_, NiCl_2_, FeCl_2_, BaCl_2_ and SrCl_2_ were from Sigma-Aldrich (Germany). Concentrated metal salts solutions (1000 mg/L) were prepared in deionized water (Millipore) and stored at 4 °C in opaque bottles. Dilutions and mixtures were freshly prepared in MilliQ water before the experiments.

### Heavy metal mixture exposure experiments

Culture conditions and heavy metal exposure of self-luminescent *Synechoccocus elongatus* PCC 7942 pBG2120 were as previously described^16^. Briefly, exposure was performed in transparent 24 well microtiter plates in 1.5 mL final volume BG11 medium (without Co, Ni, Cu, Zn). Plates were incubated at 28 °C in the light, Ca. 40 μmol photons m^2^ s^−1^ during 4 h. Luminescence measurements were performed in a Centro LB 960 luminometer in opaque white 96 well microtiter plates. The biosensor signal was expressed as luminescence induction factor (BIF) as previously reported^16^,^28^.

Mixture exposure design was adapted from^3^,^29^. Basically, *Synechoccocus elongatus* PCC 7942 pBG2120 was exposed to Zn, Cu, Cd, Ag, Co and Hg, and to their 15 possible binary combinations. Binary mixtures were prepared according to a constant ratio design (1:1) based on the individual *D*_−50_ (the dose required to get half of the MPC of each individual metal). 7 to 9 serial dilutions (factor 2) of each individual metal and binary combination were tested at the same time^4^. At least three independent experiments were performed.

### Experimental validation of the additivity definition by sham mixtures

Validation of the additivity definition was performed according to the *sham* mixture procedure^30^. sham mixtures are *false* mixtures in which the two components are exactly the same substance, and therefore, their fractional combination should be perfect additivity^30^. If an additivity formulation is correct, experimental and predicted dose-response pattern of the sham mixtures should perfectly overlap. sham mixtures of Cu:Cu, Zn:Zn and Cd:Cd (ratio 1:1) were prepared and tested as described in theory.

### Analysis of results

#### Fitting biphasic dose-response profiles and obtaining ED_p_ vectors

The entire dose-response profiles of the response of *Synechoccocus elongatus* PCC 7942 pBG2120 to the different individual heavy metal cations (Zn, Cu, Cd, Ag, Co and Hg) and their binary combinations were fitted using the non-linear functions described in Theory, with and without the use of variance-stabilizing Box-Cox transform-both-sides approach^20^. Best fit models were selected based on the minimum of the residual sum of squares^20^. *Effective doses* (*ED*_*p*_) = (*D*_*(p)*_, *E*_*(p)*_) for the different metal cations and combinations were calculated from the fitted *v-shaped* nonlinear functions.

### Additivity predictions and departures from additivity

Additive *dose-effect* profiles for the different heavy metal combinations were predicted by solving equation (7) and (9). The molar fractions (*j*_*i*_) of the mixture components were fixed according to the experimental mixture metal fractions described in Theory. Two-dimensional Combination Index *CI*_*(D, E*)*p*_ = (CI_*Dp*_, CI_*Ep*_) and weighted *CI*_*wp*_ values were computed accordingly to equations 5, 6 and 11, respectively. An illustrative example of the whole procedure of fitting dose-response curves, additivity formulation and analysis of departures from additivity can be found in Supplementary Material SM3.

### Criteria for quantification of departures from additivity

In the present work, two criteria for the definition of departures from additivity are considered. The “pharmacological criterion” considers departures from additivity when H_o_: CI = 1, is rejected (two-tailed Student’s t-Test), and is equivalent to the criterion used in human pharmacology^4^. The second criterion considered is the “risk management criterion (singlular)” which defines departure from additivity as significant based on pragmatic thresholds: Exp < 0.5 · CA (synergism) and Exp > 2 · CA (antagonism)^31^,^32^. The equivalent thresholds based on CI are: 0.5 < CI > 2. Departures from additivity for CI_*D*_ and CI_*E*_ were analyzed based on the pharmacological criterion, while analysis of departures from additivity in CI_*w*_ were based on the risk management (for consistency) criterion.

## Additional Information

**Accession codes**: Data used in the present manuscript is publicly available at: Doi: http://dx.doi.org/10.6084/m9.figshare.1476176.

**How to cite this article**: Martin-Betancor, K. *et al*. Defining an additivity framework for mixture research in inducible whole-cell biosensors. *Sci. Rep*. **5**, 17200; doi: 10.1038/srep17200 (2015).

## Supplementary Material

Supplementary Information
