# Supplementary material for: Defining an additivity framework for mixture research in inducible whole-cell biosensors
Source: Sci Rep. 2015 Nov 26;5:17200. doi: 10.1038/srep17200 (PMC4660423; doi:10.1038/srep17200)
Supplement: Supplementary Information [file srep17200-s1.pdf]

Supplementary Material:

Defining an additivity framework for mixture research in inducible whole-cell biosensors

Martin-Betancor, K[1]; Ritz, C[2]; Fernández-Piñas, F[1]; Leganés, F[1]; & Rodea-Palomares I[1]*

[1] Departament of Biology, Facultad de Ciencias, Universidad Autónoma de Madrid. 28049 Madrid, Spain.

[2] Department of Nutrition, Exercise and Sports, Faculty of Science, University of Copenhagen, Rolighedsvej 30, DK-1958 Frederiksberg C, Denmark.

*Corresponding author: Ismael Rodea-Palomares

Mailing address: Departamento de Biología, Facultad de Ciencias, Universidad Autónoma de Madrid. C/ Darwin, 2. 28049, Madrid. Spain.

Tel.: +34 914978180
Fax: +34 914978344

E-mail: ismael.rodea@uam.es

Table of contents:

Supplementary Material SM1 (Table). Summary of non-linear models and estimated parameters

Supplementary Material SM2 (Table). *EDp* vectors (-50, 0, +50) for Cu, Cd, Zn, Cu, Hg and Co.

Supplementary Material SM3 (Tutorial). Complete non-monotonic dose-response profiles and analysis of departures from additivity based on Loewe additivity and Combination Index using R.

Supplementary Material SM4 (Figure). Effect of mixture ratio for the Zn:Cd binary mixture

Supplementary Material SM5 (Table). Combination index (CI) values for the dose (*D*) and empirical effect (*E*) dimensions together with relevant statistical information for 3 selected fractional effect levels (p) of the 15 binary metal mixtures of Cu, Cd, Zn, Cu, Hg and Co.

Supplementary Material SM1 (Table). Summary of non-linear models and estimated parameters together with relevant statistical information of the model fits for the response of *Synechococcus elongatus* PCC 7942 pBG2120 to 6 heavy metals and their binary mixtures (Zn, Cu, Cd, Ag, Co and Hg)

Metal	Model Function	Parameter estimates					Residual Standard Error
			Estimate value	Std.Error	t-value	p-	
Zn	lgau2	b	0.351996	0.048207	7.301822	0.0000	0.1698429
		c	1.475965	0.597634	2.469679	0.0199	
		d	80.099960	9.894407	8.095479	0.0000	
		e	2.245102	0.043678	51.401066	0.0000	
			(2245.102)a				
		f	2.662339	0.829132	3.210994	0.0033	
Cd	lgau2	b	0.562495	0.039451	14.258093	0.0000	0.09005292
		c	1.039686	0.314707	3.303666	0.0031	
		d	49.728622	3.290085	15.114693	0.0000	
		e	5.340496	0.093375	57.193828	0.0000	
			(5340.496)[a]				
		f	2.358463	0.293418	8.037883	0.0000	
Ag	gau2	b	0.1443642	0.0093814	15.3883702	0.0000	0.08603531
		c	1.3247561	0.3296468	4.0187134	0.0007	
		d	54.6960138	3.3118291	16.5153493	0.0000	
		e	0.3292834	0.0045334	72.6353256	0.0000	
			(329.2834)[a]				
		f	3.4229734	0.4677340	7.3182051	0.0000	
Co	lgau2	b	0.532205	0.066033	8.059677	0.0000	0.07016434
		c	0.955349	0.336941	2.835362	0.0119	
		d	18.297557	1.270958	14.396664	0.0000	
		e	2.413876	0.084272	28.643759	0.0000	
			(2413.876)[a]				
		f	2.028618	0.403140	5.032046	0.0001	
Cu	lgau2	b	0.24392	0.0096496	25.277	0.0000	0.05920425
		c	0.40810	0.18429	2.2145	0.0354	
		d	43.998	1.6215	27.134	0.0000	
		e	0.046606	0.00028072	166.02	0.0000	
			(46.606)[a]				
		f	2.1294	0.13881	15.341	0.0000	
Hg	lgau2	b	0.3284459 [a]	0.0724137	4.5356872	0.0002	0.08622818
		c	0.9965343 [a]	0.5464225	1.8237431	0.0818	
		d	33.5822889 [a]	2.9519420	11.3763375	0.0000	
		e	0.0679623 [a]	0.0017048	39.8664205	0.0000	
		f	1.0307636 [a]	0.1737224	5.9333959	0.0000	
Cu:Cd	Gau2	b	0.833872	0.087886	9.488139	0.0000	0.1009751
		c	1.587702	0.559394	2.838253	0.0098	
		d	32.661863	2.571750	12.700250	0.0000	
		e	2.526442	0.039160	64.515964	0.0000	
		f	2.226572	0.326413	6.821331	0.0000	
Cu:Zn	Lgau2	b	0.210524	0.020723	10.159021	0.0000	0.03680582
		c	1.058583	0.215034	4.922875	0.0002	
		d	111.998627	5.055904	22.152047	0.0000	
		e	1.584642	0.016992	93.255607	0.0000	
		f	1.442243	0.112803	12.785522	0.0000	

Zn:Cd	Lgau2	b	0.555188	0.015215	36.489732	0.0000	0.02654068
		c	-0.290451	0.583670	-0.497628	0.626	
		d	42.910857	0.943991	45.456837	0.0000	
		e	2.321820	0.012425	186.867587	0.0000	
		f	2.024344	0.134982	14.997109	0.0000	
Cu:Hg	Lgau2	b	0.6340408	0.0362401	17.4955546	0.0000	0.05278326
		c	2.2452456	0.3135964	7.1596667	0.0000	
		d	74.6073845	3.0968760	24.0911760	0.0000	
		e	0.0260586	0.0004689	55.5735249	0.0000	
		f	3.1835757	0.3918444	8.1245914	0.0000	
Cu:Co	Gau2	b	0.85374	0.010441	81.771	0.0000	0.03405409
		c	1.3777	0.13300	10.359	0.0000	
		d	21.115	0.52473	40.239	0.0000	
		e	1.4961	0.0070987	210.75	0.0000	
		f	6.0718	0.35879	16.923	0.0000	
Co:Cd	Lgau2	b	0.26824	0.0098237	27.305	0.0000	0.05352349
		c	1.8005	0.18445	9.7614	0.0000	
		d	35.467	1.2264	28.920	0.0000	
		e	3.2161	0.013300	241.81	0.0000	
		f	2.8076	0.26146	10.738	0.0000	
Ag:Cd	Gau2	b	0.59045	0.017055	34.620	0.0000	0.0487012
		c	1.4009	0.19988	7.0088	0.0000	
		d	77.971	2.1637	36.037	0.0000	
		e	2.7331	0.0078159	349.69	0.0000	
		f	2.1606	0.079377	27.220	0.0000	
Cu:Ag	Lgau2	b	0.34083	0.013952	24.429	0.0000	0.05808011
		c	1.8282	0.24210	7.5516	0.0000	
		d	78.149	2.8113	27.798	0.0000	
		e	0.20627	0.0010609	194.43	0.0000	
		f	2.2139	0.12447	17.787	0.0000	
Zn:Ag	Gau2	b	0.28513	0.0077488	36.797	0.0000	0.04642924
		c	1.1717	0.18042	6.4942	0.0000	
		d	123.94	3.2011	38.717	0.0000	
		e	1.5822	0.0039700	398.53	0.0000	
		f	2.0776	0.061918	33.553	0.0000	
Co:Ag	Gau2	b	0.40242	0.038248	10.521	0.0000	0.04745158
		c	1.7071	0.30221	5.6487	0.0000	
		d	11.328	0.99761	11.356	0.0000	
		e	1.0509	0.0099931	105.16	0.0000	
		f	3.0655	0.74799	4.0984	0.0007	
Zn:Co	Gau2	b	0.42157	0.0097373	43.295	0.0000	0.03552789
		c	1.7071	0.30221	5.6487	0.0000	
		d	11.328	0.99761	11.356	0.0000	
		e	2.0648	0.0047810	431.88	0.0000	
		f	2.0479	0.054401	37.644	0.0000	
Cu:Hg	Lgau2	b	0.6340408	0.0362401	17.49555	0.0000	0.05278326
		c	2.2452456	0.3135964	7.1596667	0.0000	
		d	74.6073845	3.0968760	24.0911760	0.0000	
		e	0.0260586	0.0004689	55.5735249	0.0000	
		f	3.1835757	0.3918444	8.1245914	0.0000	
Cd:Hg	Lgau2	b	0.335736	0.010892	30.824749	0.0000	0.03659033
		c	1.487436	0.158866	9.362838	0.0000	
		d	71.957893	1.550272	46.416307	0.0000	
		e	2.487738	0.017196	144.672177	0.0000	

		f	2.508632	0.142398	17.617043	0.0000	
Zn:Hg	Gau2	b	0.28384	0.015798	17.967	0.0000	0.07234654
		c	1.3458	0.29692	4.5326	0.0001	
		d	154.63	8.0164	1.9290	0.0000	
		e	1.3592	0.0060743	223.76	0.0000	
		f	2.2107	0.13004	16.999	0.0000	
Hg:Co	Lgau2	b	0.464864	0.054759	8.489311	0.0000	0.05336395
		c	2.154873	0.322521	6.681348	0.0000	
		d	65.070756	2.618477	24.850612	0.0000	
		e	0.754579	0.030447	24.783295	0.0000	
		f	2.812375	0.814988	3.450817	0.0000	
Hg:Ag	Gau2	b	0.071195	0.0014252	49.954	0.0000	0.02301
		c	-0.0050033	0.12298	-0.040684	0.968	
		d	34.655	0.58610	59.128	0.0000	
		e	0.18673	0.00079776	234.07	0.0000	
		f	1.9507	0.062492	31.215	0.0000	

[a] (nM)

gau and lgau refers to the Normal and log-normal biphasic dose-response models described in the gaussian {drc} functions in *drc* R package [1]. Parameter estimates and estimated residual standard errors are calculated using the function drm {drc} which is based on the function optim {stats} [2] which relies on the minimisation of the minus log likelihood function. For a quantitative response this reduces to least squares estimation, which is carried out by minimising the following sums of squares

$$\sum_{i=1}^N [w_i (y_i-f_i)]^2$$

where y_i, f_i, and w_i correspond to the observed value, expected value, and the weight respectively, for the ith observation (from 1 to N).

Supplementary Material SM2 (Table). *EDp* vectors (-50, 0, +50) for Cu, Cd, Zn, Cu, Hg and Co.

Metal	Fractional effect	*EDp* (µM, BIF) D(*p*) (µM)	E(*p*) (BIF)
Zn	ED_{-50}	1.508±0.082	40.79±3.33
	ED_{0}	2.8±0.11	79.31±8.89
	ED_{+50}	3.34±0.18	4.79±7.38
Cd	ED_{-50}	2.8±0.11	25.38±1.69
	ED_{0}	5.94±0.18	49.24±3.03
	ED_{+50}	10.19±0.39	25.38±1.49
Ag	ED_{-50}	0.17±0.006	28.01±1.79
	ED_{0}	0.37±0.013	54.16±3.12
	ED_{+50}	0.49±0.012	28.01±4.39
Co	ED_{-50}	1.29±0.07	9.63±15
	ED_{0}	2.61±0.1	18.12±1.17
	ED_{+50}	4.51±0.24	9.63±0.76
Cu	ED_{-50}	0.035±0.0004	22.2±0.97
	ED_{0}	0.048±0.0003	43.56±1.53
	ED_{+50}	0.062±0.0007	22.2±0.95
Hg[a]	ED_{-50}	0.043±0.003	17.29±1.24
	ED_{0}	0.068±0.001	33.25±3.04
	ED_{+50}	0.11±0.008	17.29±1.08

±: Standard error. Standard error are estimated from the non-linear fitted model. Fitted non-linear models and estimated parameters can be found in Table SM1.

[a] (nM).

Supplementary Material SM3 (Tutorial)

Fitting non-monotonic dose-response with differential maximal effects and analysis of departures from additivity based on multivariate Loewe additivity using R.

From: *Defining an additivity framework for mixture research in inducible whole-cell biosensors.*

Martin-Betancor, K[1]; Ritz, C[2]; Fernández-Piñas, F[1]; Leganés, F[1]; & Rodea-Palomares I[1]*

[1] Departament of Biology, Facultad de Ciencias, Universidad Autónoma de Madrid. 28049 Madrid, Spain.

[2] Department of Nutrition, Exercise and Sports, Faculty of Science, University of Copenhagen, Rolighedsvej 30, DK-1958 Frederiksberg C, Denmark.

*Corresponding author: Ismael Rodea-Palomares

Mailing address: Departamento de Biología, Facultad de Ciencias, Universidad Autónoma de Madrid. C/ Darwin, 2. 28049, Madrid. Spain.

Tel.: +34 914978180
Fax: +34 914978344

E-mail: ismael.rodea@uam.es

A novel additivity framework for mixture-effect research in the framework of whole-cell inducible biosensors has been mathematically developed. The method proposes a multivariative extension of the effective dose (*EDp*) notation in order to take into account the occurrence of differential maximal effects and inhibition beyond the maximum permissive concentrations (MPCs). This allow a dimensional extension of Loewe additivity which enables its direct application in a biphasic dose-response framework. The utilities that allow to achieve this new approach have been incorporated in the (*drc*) package for R[1].

#Example data:

The dataset "metaldata.csv" and the functional R-script "SM3_Script_R.R" used in the present example are freely available from http://dx.doi.org/10.6084/m9.figshare.1476176

In the present tutorial, we will explain how to use the novel method developed in order to model dose-response profiles of an inducible whole cell biosensor to individual *stimuli* and mixtures (in the present case, different heavy metals). We will analyse the "metaldata.csv" dataset. This dataset includes the raw data resulted from the individual exposure of the inducible whole cell biosensor *Synecchoccocus elongatus* PCC 7942 pBG2120[3] to six metals and their 15 possible binary combinations.

The dataset is importable as data.frame in R as follows: (from the root directory:

metaldata<-read.csv(file=”metaldata.csv”)

The data frame have 3 variables: "metal" (factor), "conc" (numeric), "IR" (induction factor, the same asBIF in the main text) (numeric). In the tutorial, we will explain the *drc* extension using the data for Zn, Cd and its mixture, ZnCd.

#Make a subset with the Zn data

```
Zn <- metaldata[metaldata$metal=="Zn",]

Zn
```

```
   metal    conc        IR
1     Zn  0.0369  0.9142857
2     Zn  0.0369  0.9756098
3     Zn  0.0369  0.8974359
4     Zn  0.0925  0.9523810
5     Zn  0.0925  0.8780488
6     Zn  0.0925  1.1666667
7     Zn  0.1859  0.9523810
8     Zn  0.1859  1.1707317
9     Zn  0.1859  1.2115385
10    Zn  0.5693  1.7523810
11    Zn  0.5693  1.6585366
12    Zn  0.5693  1.7948718
13    Zn  0.9684  5.9809524
14    Zn  0.9684  4.6341463
15    Zn  0.9684  4.8461538
16    Zn  1.3836 14.2857143
17    Zn  1.3836 19.7073171
18    Zn  1.3836 22.7051282
19    Zn  1.4472 43.9500000
20    Zn  1.4472 44.0869565
21    Zn  1.4472 43.2000000
22    Zn  2.0371 67.1238095
23    Zn  2.0371 74.0975610
24    Zn  2.0371 86.0192308
25    Zn  3.2111 31.1619048
26    Zn  3.2111 61.2195122
27    Zn  3.2111 64.6153846
28    Zn  4.4946  3.8857143
29    Zn  4.4946  4.8292683
30    Zn  4.4946  5.3846154
31    Zn 10.7532  0.3428571
32    Zn 10.7532  0.5853659
33    Zn 10.7532  0.6730769
```

#Fitting biphasic dose-response profiles:

Inducible whole cell biosensors usually present inverted v-shaped biphasic dose-response profiles. This specific type of dose-response pattern may be fitted using nonlinear regression model equations as Gaussian (gau) or LogGaussian (lgau) with or without the use of variance-stabilizing Box-Cox transform-both-sides approach. (For more detail see section *2.1 Fitting biphasic dose-response profiles* of the manuscript).

#gaussian function:

```
Zn.gau <- drm(IR~conc, data=Zn, fct=gaussian(), na.action=na.omit)

summary(Zn.gau)
```

```
Model fitted: Gaussian (5 parms)

Parameter estimates:

              Estimate Std. Error    t-value p-value
b:(Intercept) 8.8613e-01 9.3682e-03 9.4589e+01  0.0000
c:(Intercept) 2.1660e+00 1.2821e+00 1.6895e+00  0.1022
d:(Intercept) 7.5746e+01 3.3920e+00 2.2331e+01  0.0000
e:(Intercept) 2.3418e+00 8.6316e-03 2.7131e+02  0.0000
```

```
f:(Intercept) 1.3880e+01 2.8981e+00 4.7894e+00  0.0000

Residual standard error:

 5.87511 (28 degrees of freedom)
```

For complete information of the drm {drc} consult the tutorial of drc package http://cran.r-project.org/web/packages/drc/index.html

# Model checking

Best-fit models can be selected based on the residual standard error that is indicated in the model summary. For vidual observation they can be plotted:

```
plot(fitted(Zn.gau), residuals(Zn.gau), ylim = c(-20, 20))
```

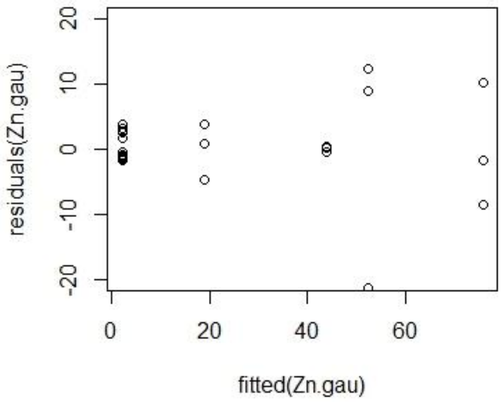

# Gaussian function with Box-Cox transform

```
Zn.gau2 <- drm(IR~conc, data=Zn, fct=gaussian(), na.action=na.omit, bcVal = 0, bcAdd = 10)
summary(Zn.gau2)
```

```
Model fitted: Gaussian (5 parms)

Parameter estimates:

              Estimate Std. Error   t-value p-value
b:(Intercept)  0.793259   0.075520 10.503961  0.0000
c:(Intercept)  1.613775   0.500908  3.221697  0.0032
d:(Intercept) 81.080825  10.982114  7.382989  0.0000
e:(Intercept)  2.422227   0.042576 56.892278  0.0000
f:(Intercept)  3.115795   0.624967  4.985533  0.0000

Residual standard error:

 0.17677 (28 degrees of freedom)

Non-normality/heterogeneity adjustment through optimal Box-Cox transformation

Specified lambda: 0
```

# Model checking

```
plot(fitted(Zn.gau2), residuals(Zn.gau2), ylim = c(-0.4, 0.4))
```

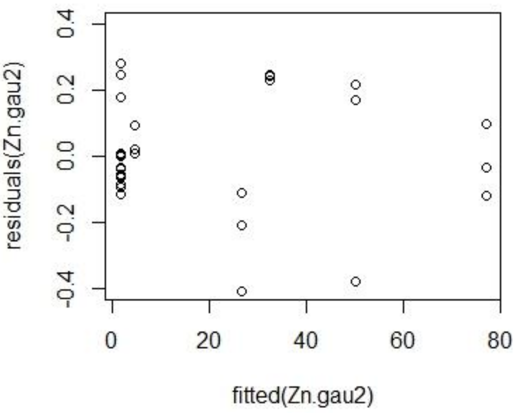

#lgaussian function

```
Zn.lgau <- drm(IR~conc, data=Zn, fct=lgaussian(), na.action=na.omit)

summary(Zn.lgau)
```

```
Model fitted: Log-Gaussian (5 parms)

Parameter estimates:

               Estimate Std. Error    t-value p-value
b:(Intercept) 4.0134e-01 6.4603e-03 6.2124e+01  0.0000
c:(Intercept) 2.1661e+00 1.2821e+00 1.6895e+00  0.1022
d:(Intercept) 7.5747e+01 3.3920e+00 2.2331e+01  0.0000
e:(Intercept) 2.1746e+00 1.2839e-02 1.6938e+02  0.0000
f:(Intercept) 9.1066e+00 1.8773e+00 4.8510e+00  0.0000

Residual standard error:

 5.87511 (28 degrees of freedom)
```

#Model checking

```
plot(fitted(Zn.lgau), residuals(Zn.lgau), ylim = c(-20, 20))
```

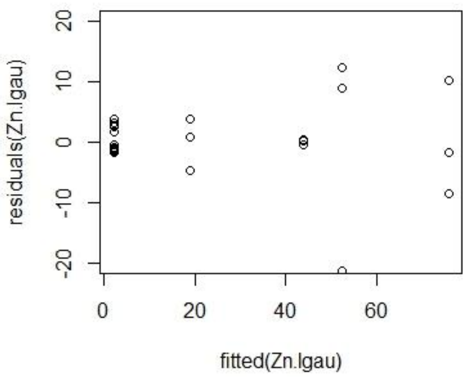

# lgaussian function with Box-Cox transform

```
Zn.lgau2 <- drm(IR~conc, data=Zn, fct=lgaussian(), na.action=na.omit, bcVal = 0, bcAdd = 10)
summary(Zn.lgau2)
```

```
Model fitted: Log-Gaussian (5 parms)

Parameter estimates:

              Estimate Std. Error   t-value p-value
b:(Intercept)  0.351996   0.048207  7.301822  0.0000
c:(Intercept)  1.475965   0.597634  2.469679  0.0199
d:(Intercept) 80.099960   9.894407  8.095479  0.0000
e:(Intercept)  2.245102   0.043678 51.401066  0.0000
f:(Intercept)  2.662339   0.829132  3.210994  0.0033

Residual standard error:

 0.1698429 (28 degrees of freedom)

Non-normality/heterogeneity adjustment through optimal Box-Cox transformation

Specified lambda: 0
```

#Model checking

```
plot(fitted(Zn.lgau2), residuals(Zn.lgau2), ylim = c(-0.4, 0.4))
```

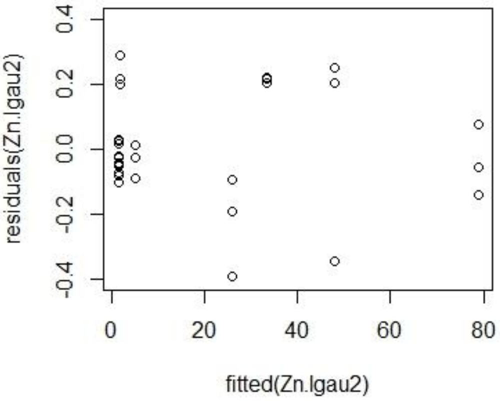

#plot:

We can visually observe all the functions used in order to have a visual overview of the models fitted.

```
plot(Zn.gau, type = "obs", col= "black", log = "")
plot(Zn.gau, type = "none", add = TRUE, col = "red")
plot(Zn.gau2, type = "none", add = TRUE, col = "yellow")
plot(Zn.lgau, type = "none", add = TRUE, col = "blue")
plot(Zn.lgau2, type = "none", add = TRUE, col = "green")
```

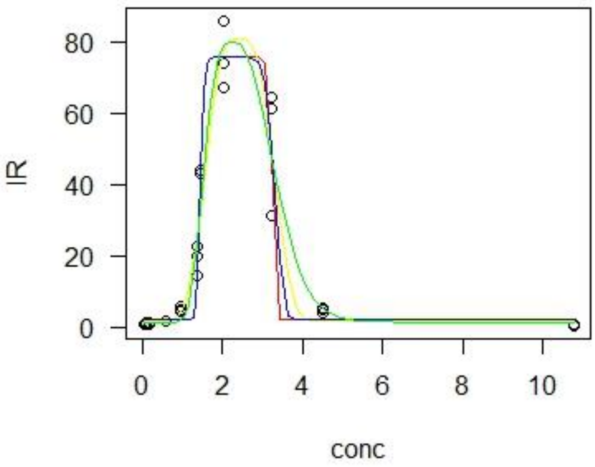

Based on the residuals standard errors and the visual examination, we can conclude that the LogGaussian with Box-Cox modification (green line) is the model that fit better the experimental data, therefore it will be used for fitting Zn response in the rest of calculations.

#Effective doses (*EDp*) calculation:

Using the *ED* {*drc*} function, we can get the *D(p)* vectors at any desired fractional effect *p*. We selected the three representative *p* levels: -50 (half of the *Emax*, in the induction region), 99.9 (*Emax*) and +50 (half of the *Emax*, in the inhibition region) (*Equivalent to the -50, 0, +50 p levels in the manuscript*). For more details, see section *2.2 a multivariate extension of the effective dose notation* of the manuscript). Note: to get the *E*(*p*) values, for the moment it is required to use the ***indicesFct*** {*drc*} function which will be described in next sections.

```
ED(Zn.lgau2, -50, interval = "delta", bound = FALSE)
```

```
     Estimate Std. Error    Lower  Upper
1:-50 1.508038   0.082849 1.338329 1.6777
```

```
ED(Zn.lgau2, 99.9,interval = "delta")
```

```
      Estimate Std. Error   Lower  Upper
1:99.9  2.32300    0.10458 2.10877 2.5372
```

```
ED(Zn.lgau2, 50, interval = "delta")
```

```
    Estimate Std. Error   Lower  Upper

1:50  3.34241    0.18363 2.96627 3.7186
```

#Cd fit:

Now, we are going to fit the second data component, in that case, Cd. For that, we use the same steps as for Zn.

#Make a subset with the Cd data

```
Cd <- metaldata[metaldata$metal=="Cd",]
```

```
Cd
```

#Fitting biphasic dose-response profiles:

#gaussian function

```
Cd.gau <- drm(IR~conc, data=Cd, fct=gaussian(), na.action=na.omit)
```

```
summary(Cd.gau)
```

```
Model fitted: Gaussian (5 parms)

Parameter estimates:

              Estimate Std. Error   t-value p-value
b:(Intercept)  4.079689   0.115139 35.432648  0.0000
c:(Intercept)  1.283627   1.275138  1.006657  0.3246
d:(Intercept) 42.981885   1.907644 22.531393  0.0000
e:(Intercept)  6.850150   0.070015 97.838717  0.0000
f:(Intercept)  7.805005   3.237173  2.411056  0.0243

Residual standard error:

 4.4849 (23 degrees of freedom)
```

#Model checking

```
plot(fitted(Cd.gau), residuals(Cd.gau), ylim = c(-10, 10))
```

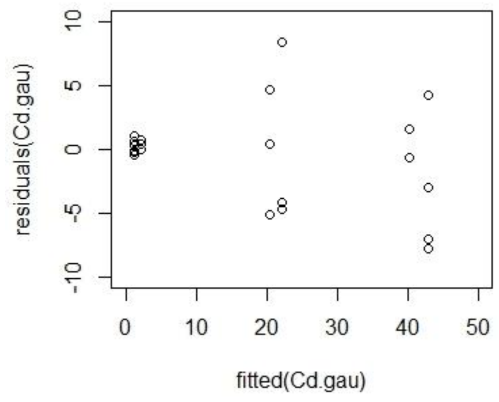

#Gaussian function with Box-Cox transform

```
Cd.gau2 <- drm(IR~conc, data=Cd, fct=gaussian(), na.action=na.omit, bcVal = 0, bcAdd = 10)
summary(Cd.gau2)
```

```
Model fitted: Gaussian (5 parms)

Parameter estimates:

               Estimate Std. Error    t-value p-value
b:(Intercept)   4.056422   0.090069  45.036766  0.0000
c:(Intercept)   1.202271   0.352358   3.412072  0.0024
d:(Intercept)  42.700145   2.340528  18.243805  0.0000
e:(Intercept)   6.851570   0.053432 128.230478  0.0000
f:(Intercept)   7.234677   1.057973   6.838241  0.0000

Residual standard error:

 0.1088791 (23 degrees of freedom)

Non-normality/heterogeneity adjustment through optimal Box-Cox transformation

Specified lambda: 0
```

#Model checking

```
plot(fitted(Cd.gau2), residuals(Cd.gau2), ylim = c(-0.2, 0.2))
```

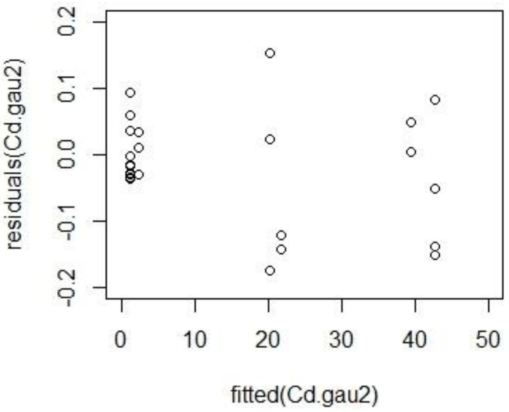

#lgaussian function

```
Cd.lgau <- drm(IR~conc, data=Cd, fct=lgaussian(), na.action=na.omit)

summary(Cd.lgau)
```

```
Model fitted: Log-Gaussian (5 parms)

Parameter estimates:

              Estimate Std. Error   t-value p-value
b:(Intercept)  0.544683   0.032578 16.719520  0.0000
c:(Intercept)  0.806563   0.920847  0.875893  0.3901
d:(Intercept) 51.009281   2.143174 23.800808  0.0000
e:(Intercept)  5.315768   0.093498 56.854176  0.0000
f:(Intercept)  2.105116   0.261655  8.045382  0.0000

Residual standard error:

 3.086809 (23 degrees of freedom)
```

#Model checking

```
plot(fitted(Cd.lgau), residuals(Cd.lgau), ylim = c(-6, 6), xlim = c(0, 50))
```

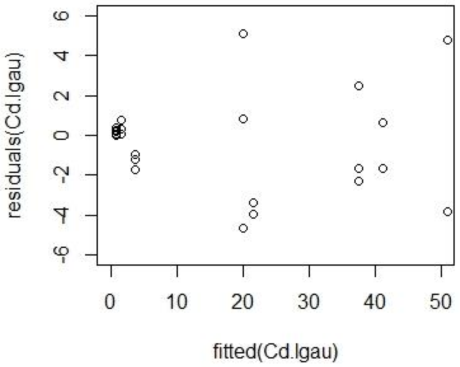

#lgaussian function with Box-Cox transform

```
Cd.lgau2 <- drm(IR~conc, data=Cd, fct=lgaussian(), na.action=na.omit, bcVal = 0, bcAdd = 10)

summary(Cd.lgau2)
```

```
Model fitted: Log-Gaussian (5 parms)

Parameter estimates:

               Estimate Std. Error   t-value p-value
b:(Intercept)  0.562495   0.039451 14.258093  0.0000
c:(Intercept)  1.039686   0.314707  3.303666  0.0031
d:(Intercept) 49.728622   3.290085 15.114693  0.0000
e:(Intercept)  5.340496   0.093375 57.193828  0.0000
f:(Intercept)  2.358463   0.293418  8.037883  0.0000

Residual standard error:

 0.09005292 (23 degrees of freedom)

Non-normality/heterogeneity adjustment through optimal Box-Cox transformation

Specified lambda: 0
```

#Model checking

```
plot(fitted(Cd.lgau2), residuals(Cd.lgau2), ylim = c(-0.3, 0.3))
```

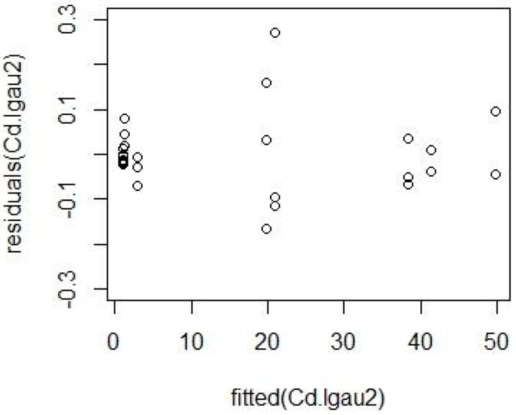

#plot:

```
plot(Cd.gau, type = "obs", col= "black", log = "", ylim = c(0, 60))
plot(Cd.gau, type = "none", add = TRUE, col = "red")
plot(Cd.gau2, type = "none", add = TRUE, col = "yellow")
plot(Cd.lgau, type = "none", add = TRUE, col = "blue")
plot(Cd.lgau2, type = "none", add = TRUE, col = "green")
```

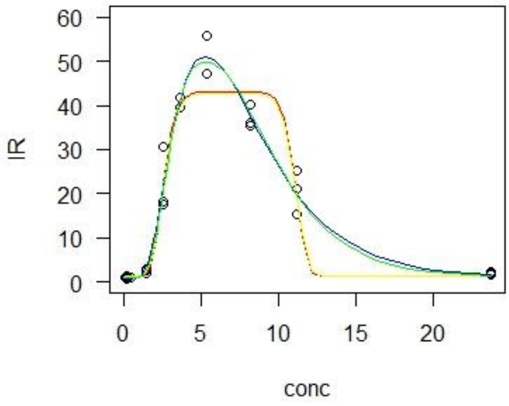

Based on the residuals standard errors and the visual examination, we can observed that the LogGaussian with Box-Cox modification (green line) is the model that fits better Cd experimental data, therefore it will be used for fitting Cd response in the rest of calculations.

#Effective doses (*D(p)*) calculation:

```
ED(Cd.lgau2, -50, interval = "delta", bound = FALSE)
```

```
   Estimate Std. Error   Lower  Upper
1:-50  2.79902    0.10714 2.57737 3.0207
```

```
ED(Cd.lgau2, 99.9,interval = "delta")
```

```
      Estimate Std. Error   Lower  Upper
1:99.9  5.56039    0.11333 5.32594 5.7948
```

```
ED(Cd.lgau2, 50,interval = "delta")
```

```
    Estimate Std. Error    Lower  Upper
1:50 10.18961    0.39005  9.38273 10.996
```

#Fitting mixture data:

In order to predict the biosensor response to any combination of the individual metals and also, to be able to analyse departures from additivity, it is required to fit the mixture data to a nonlinear regression model equation. Zn and Cd mixture data are used as example.

# Make a subset with the ZnCd data

```
ZnCd <- metaldata[metaldata$metal=="ZnCd",]
```

#gaussian function

```
ZnCd.gau <- drm(IR~conc, data=ZnCd, fct=gaussian(), na.action=na.omit)

summary(ZnCd.gau)
```

```
Model fitted: Gaussian (5 parms)

Parameter estimates:

                Estimate Std. Error    t-value p-value
b:(Intercept) 9.1892e-03         NA         NA      NA
c:(Intercept) 2.3311e+00 1.1228e+00 2.0760e+00  0.0555
d:(Intercept) 1.0401e+03         NA         NA      NA
e:(Intercept) 2.8890e+00 2.3606e-02 1.2239e+02  0.0000
f:(Intercept) 4.1168e-01 1.6657e-02 2.4716e+01  0.0000

Residual standard error:

 2.123171 (15 degrees of freedom)
```

#Model checking

```
plot(fitted(ZnCd.gau), residuals(ZnCd.gau))
```

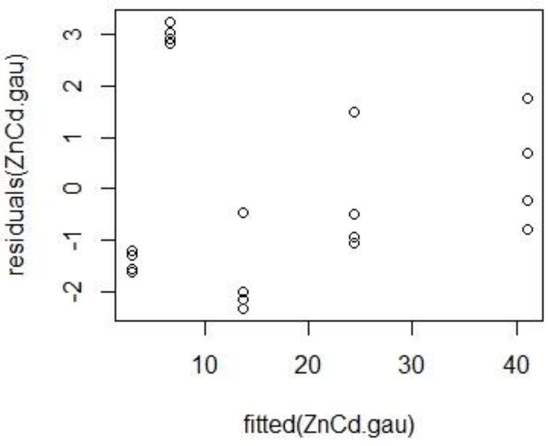

#Gaussian function with Box-Cox transform

```
ZnCd.gau2 <- drm(IR~conc, data=ZnCd, fct=gaussian(), na.action=na.omit, bcVal = 0, bcAdd = 10)

summary(ZnCd.gau2)
```

```
Model fitted: Gaussian (5 parms)

Parameter estimates:

                Estimate Std. Error    t-value p-value
b:(Intercept)   0.040441   0.032399   1.248238  0.2311
c:(Intercept)   0.561210   1.249161   0.449269  0.6597
d:(Intercept) 330.288516 136.118307   2.426481  0.0283
e:(Intercept)   2.899675   0.049022  59.151001  0.0000
f:(Intercept)   0.473996   0.074228   6.385670  0.0000

Residual standard error:

 0.1078746 (15 degrees of freedom)

Non-normality/heterogeneity adjustment through optimal Box-Cox transformation

Specified lambda: 0
```

#Model checking

```
plot(fitted(ZnCd.gau2), residuals(ZnCd.gau2), ylim = c(-0.15, 0.15))
```

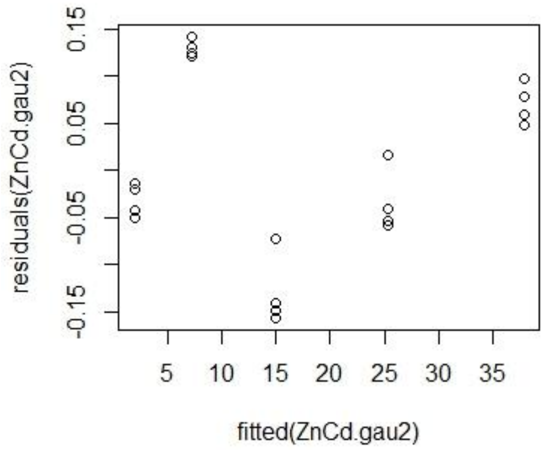

#lgaussian function

```
ZnCd.lgau <- drm(IR~conc, data=ZnCd, fct=lgaussian(), na.action=na.omit)
summary(ZnCd.lgau)
```

```
Model fitted: Log-Gaussian (5 parms)

Parameter estimates:

              Estimate Std. Error    t-value p-value
b:(Intercept)   0.555663   0.015419  36.038564  0.0000
c:(Intercept)  -0.274800   1.032831  -0.266065  0.7938
d:(Intercept)  42.904780   0.747360  57.408441  0.0000
e:(Intercept)   2.321479   0.018413 126.077969  0.0000
f:(Intercept)   2.028462   0.164444  12.335300  0.0000

Residual standard error:

 0.834502 (15 degrees of freedom)
```

#Model checking

```
plot(fitted(ZnCd.lgau), residuals(ZnCd.lgau), ylim = c(-2, 2))
```

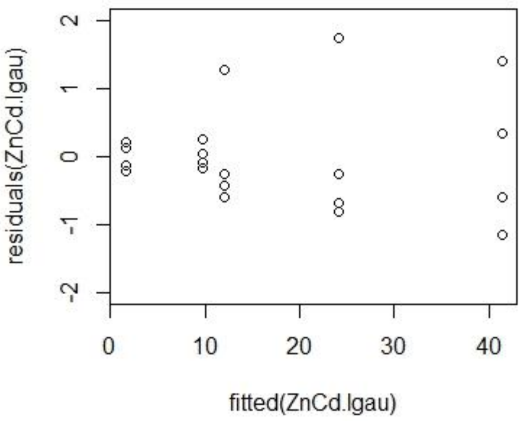

#lgaussian function with Box-Cox transform

```
ZnCd.lgau2 <- drm(IR~conc, data=ZnCd, fct=lgaussian(), na.action=na.omit, bcVal = 0, bcAdd = 10)

summary(ZnCd.lgau2)
```

```
Model fitted: Log-Gaussian (5 parms)

Parameter estimates:

               Estimate Std. Error    t-value p-value
b:(Intercept)   0.555188   0.015215  36.489732   0.000
c:(Intercept)  -0.290451   0.583670  -0.497628   0.626
d:(Intercept)  42.910857   0.943991  45.456837   0.000
e:(Intercept)   2.321820   0.012425 186.867587   0.000
f:(Intercept)   2.024344   0.134982  14.997109   0.000

Residual standard error:

 0.02654068 (15 degrees of freedom)

Non-normality/heterogeneity adjustment through optimal Box-Cox transformation

Specified lambda: 0
```

#Model checking

```
plot(fitted(ZnCd.lgau2), residuals(ZnCd.lgau2), ylim = c(-0.06, 0.06))
```

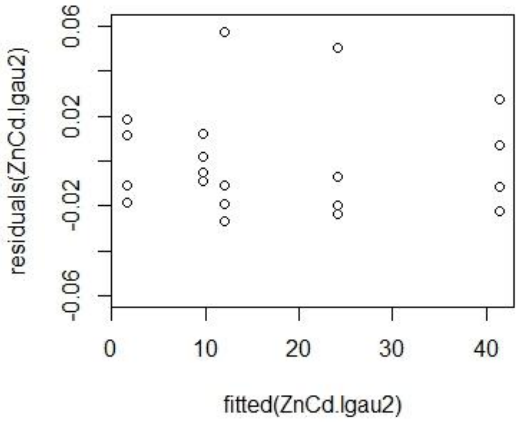

#Plot:

```
plot(ZnCd.gau, type = "obs", col= "black", ylim = c(0, 300))
plot(ZnCd.gau, type = "none", add = TRUE, col = "red")
plot(ZnCd.gau2, type = "none", add = TRUE, col = "yellow")
plot(ZnCd.lgau, type = "none", add = TRUE, col = "blue")
plot(ZnCd.lgau2, type = "none", add = TRUE, col = "green")
```

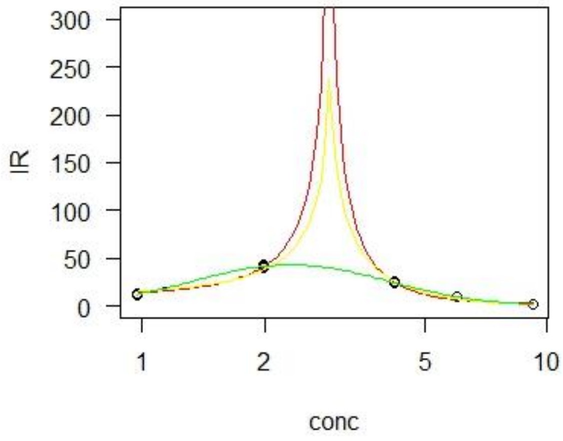

Based on the residuals standard errors and visual observation, we can infer that the LogGaussian with Box-Cox modification (green line) is the model that fits better the experimental data, therefore it will be used for fitting ZnCd response in the rest of calculations.

#Effective doses (*D(p)*) calculation:

```
ED(ZnCd.lgau2, -50, interval = "delta", bound = FALSE)
```

```
    Estimate Std. Error    Lower  Upper
1:-50 1.209189   0.014632 1.178001 1.2404
```

```
ED(ZnCd.lgau2, 99.9,interval = "delta")
```

```
    Estimate Std. Error    Lower  Upper
1:99.9 2.382456   0.015367 2.349703 2.4152
```

```
ED(ZnCd.lgau2, 50,interval = "delta")
```

```
  Estimate Std. Error    Lower  Upper
1:50 4.458236   0.053948 4.343248 4.5732
```

#Additivity predictions and departures from additivity:

#*E(p)* calculation

We apply the method to perform additivity predictions and to study the nature of the interaction through the ***indicesFct*** {drc} command. The first datum that has to be indicated is the ratio in which metals are present in the mixture. After that, we have to introduce the list with the model equations fitted for the mixture and the individual metals, respectively. The ratio only has to be indicated for the individual metal indicated first. (For example, in the case below, the ratio Zn:Cd is 0.355:0.645, therefore, as Zn is the first individual metal introduced, we indicated is 0.355 as ratio data). In c() vector we indicated the fractional effect (*p*) which we want to be considered.

```
indicesFct(0.355,list(ZnCd.lgau2, Zn.lgau2, Cd.lgau2), c(-0.2, -0.5, -1, -2, -5, -10, -20, -30, -40,
-50, -60, -70, -80, -90, -99, 99, 80, 70, 60, 50, 40, 30, 20, 10, 5, 2, 1, 0.5, 0.2))
```

#ECmat: Matrix with the fitted nonlinear regression model equations concentration data for the mixture (ECmix, Zn:Cd), the first individual metal indicated (EC1, Zn) and the second individual metal indicated (EC2, Cd) and the standard error, respectively. These data correspond to the D*(p)* data for all the fractional effect indicated in indicesFct.

#Emat: Matrix with the fitted nonlinear regression model equations effect data for the mixture (ECmix, Zn:Cd), the first individual metal indicated (EC1, Zn) and the second individual metal indicated (EC2, Cd) and the standard error, respectively. These data correspond to the E*(p)* data for all the fractional effect indicated.

```
#ECmat
```

```
$ECmat
        ECmix       EC1       EC2      SEmix        SE1        SE2
-0.2  0.3377212 0.9063974  1.038593 0.04011755 0.13046075 0.14144360
-0.5  0.3908416 0.9554918  1.155985 0.03941034 0.11453523 0.13457294
-1    0.4403353 0.9982981  1.262736 0.03821894 0.10068741 0.12756483
-2    0.5008234 1.0475599  1.390481 0.03621944 0.08504322 0.11864606
-5    0.6052838 1.1265598  1.605791 0.03175981 0.06167670 0.10360652
-10   0.7130718 1.2020991  1.823015 0.02636580 0.04433297 0.09065132
-20   0.8635123 1.3004321  2.121074 0.01865975 0.03990628 0.08155946
-30   0.9854817 1.3758876  2.360446 0.01376951 0.05191294 0.08431427
```

```
-40   1.0980212 1.4431499  2.580827 0.01236843 0.06724096 0.09380770
-50   1.2091890 1.5080376  2.799017 0.01463218 0.08284889 0.10714382
-60   1.3247947 1.5744593  3.027361 0.01900860 0.09824789 0.12269719
-70   1.4513381 1.6465755  3.280125 0.02418490 0.11340309 0.13948356
-80   1.5994438 1.7311354  3.581510 0.02937550 0.12816148 0.15641649
-90   1.7951676 1.8452853  3.993509 0.03323070 0.14111191 0.17001128
-99   2.1420593 2.0701401  4.797048 0.02491718 0.13005296 0.14361250
99    2.5166671 2.4348518  5.945510 0.02927474 0.15296534 0.17799479
80    3.3704531 2.9116638  7.963371 0.06190199 0.21555976 0.34778696
70    3.7144001 3.0611923  8.695064 0.06189626 0.21083069 0.36974764
60    4.0691968 3.2014066  9.421043 0.05838621 0.19977109 0.38182950
50    4.4582362 3.3424130 10.189613 0.05394832 0.18362621 0.39004910
40    4.9096050 3.4926962 11.051070 0.05530320 0.16273586 0.40168347
30    5.4702694 3.6634419 12.082839 0.07643259 0.13822354 0.43159454
20    6.2429337 3.8760073 13.446443 0.13490439 0.11894280 0.51704222
10    7.5600385 4.1930688 15.644902 0.27953208 0.15463881 0.77795888
5     8.9063184 4.4742271 17.761275 0.46732288 0.24495419 1.14596724
2    10.7639755 4.8116429 20.511540 0.77844851 0.39061976 1.75019587
1    12.2426021 5.0490772 22.586587 1.06259775 0.50924519 2.28175498
0.5  13.7929269 5.2752772 24.672378 1.39080342 0.63234981 2.87221259
0.2  15.9624260 5.5610094 27.461096 1.89615994 0.80041434 3.73986448
```

```
$Emat
          Emix        E1        E2     SEmix       SE1       SE2
-0.2 -0.2040480  1.633213  1.137063 0.5205959 0.4644456 0.2752171
-0.5 -0.0744441  1.869085  1.283130 0.4604050 0.7311427 0.2751321
-1    0.1415624  2.262205  1.526575 0.3871991 1.2358878 0.3226767
-2    0.5735755  3.048445  2.013464 0.2880300 2.0127812 0.4429080
-5    1.8696147  5.407164  3.474132 0.1717987 3.3361324 0.6960765
-10   4.0296801  9.338364  5.908579 0.2052600 4.1595662 0.8915007
-20   8.3498109 17.200764 10.777473 0.2567516 4.0115727 1.0526113
-30  12.6699416 25.063163 15.646367 0.3034367 3.2581518 1.2316369
-40  16.9900724 32.925563 20.515260 0.4120100 2.9174574 1.4676017
-50  21.3102031 40.787962 25.384154 0.5412729 3.3301463 1.6940712
-60  25.6303338 48.650362 30.253047 0.6439274 4.0714852 1.8533158
-70  29.9504646 56.512761 35.121941 0.6877660 4.7374010 1.9219699
-80  34.2705953 64.375161 39.990835 0.6544011 5.2893357 1.9432093
-90  38.5907261 72.237560 44.859728 0.5856322 6.2821053 2.1387117
-99  42.4788438 79.313720 49.241733 0.8282188 9.1805150 3.0337516
99   42.4788438 79.313720 49.241733 0.8505941 8.8935420 3.0498938
80   34.2705953 64.375161 39.990835 0.5437934 7.9318629 1.6544342
70   29.9504646 56.512761 35.121941 0.5390104 8.5289396 1.5638688
60   25.6303338 48.650362 30.253047 0.4828725 8.3180725 1.5340835
50   21.3102031 40.787962 25.384154 0.3876131 7.3784991 1.4919847
40   16.9900724 32.925563 20.515260 0.2912417 5.9051575 1.4375273
30   12.6699416 25.063163 15.646367 0.2511280 4.2123056 1.3878589
20    8.3498109 17.200764 10.777473 0.2655813 2.9172177 1.3277733
10    4.0296801  9.338364  5.908579 0.2112043 2.6130773 1.1303774
5     1.8696147  5.407164  3.474132 0.1460100 2.2864276 0.8458768
2     0.5735755  3.048445  2.013464 0.2697191 1.4925741 0.5097317
1     0.1415624  2.262205  1.526575 0.3772735 0.9548770 0.3522130
0.5  -0.0744441  1.869085  1.283130 0.4551203 0.6017063 0.2840370
0.2  -0.2040480  1.633213  1.137063 0.5183190 0.4483813 0.2753855
```

#CAx: data frame including a series of relevant parameters from the mixture models:

CAx$ComInd is the Combination Index calculated for the dose dimension for each p level, CAx $seCI is the standard errors of the combination index, CAx$Diff is the difference from 1 of the C ombination index, CA$CAdiffp: p-value of the two tailed Student T-test of the Null hypothesis C I =1. CAx$PredAdd is the prediction of the dose (*D*) required to produce each p level under the h ypothesis of additivity, and CAx$sePredAdd is the standard error of the dose (*D*) required to pro duce each p level under the hypothesis of additivity.

#CAy: The same that **#CAx** but for the empirical effect (E) axis.

CAx

	combInd	seCI	CAdiff	ciloCAdiff	cihiCAdiff	CAdiffp	PredAdd	sePredAdd
-0.2	0.3420080	0.05318707	-0.65799204	0.2377613	0.4462546	0.000000e+00	0.987466	0.09911044
-0.5	0.3632882	0.04784768	-0.63671184	0.2695067	0.4570696	0.000000e+00	1.075845	0.09115646
-1	0.3815069	0.04315301	-0.61849311	0.2969270	0.4660868	0.000000e+00	1.154200	0.08371682
-2	0.4020365	0.03779096	-0.59796346	0.3279663	0.4761068	0.000000e+00	1.245716	0.07480133
-5	0.4338613	0.02955270	-0.56613872	0.3759380	0.4917846	0.000000e+00	1.395109	0.06059542
-10	0.4628736	0.02259682	-0.53712644	0.4185838	0.5071633	0.000000e+00	1.540533	0.04910660
-20	0.4983135	0.01643862	-0.50168648	0.4660938	0.5305332	0.000000e+00	1.732870	0.04319287
-30	0.5235555	0.01542966	-0.47644447	0.4933134	0.5537977	0.000000e+00	1.882287	0.04884191
-40	0.5445192	0.01718988	-0.45548075	0.5108271	0.5782114	0.000000e+00	2.016497	0.05946825
-50	0.5632927	0.02011918	-0.43670733	0.5238591	0.6027263	0.000000e+00	2.146644	0.07213750
-60	0.5809637	0.02340490	-0.41903632	0.5350901	0.6268373	0.000000e+00	2.280340	0.08584242
-70	0.5982965	0.02666656	-0.40170354	0.5460300	0.6505629	0.000000e+00	2.425784	0.10027830
-80	0.6160407	0.02959570	-0.38395927	0.5580332	0.6740483	0.000000e+00	2.596328	0.11525769
-90	0.6352995	0.03143490	-0.36470046	0.5736871	0.6969119	0.000000e+00	2.825703	0.12966401
-99	0.6553495	0.02578785	-0.34465050	0.6048053	0.7058937	0.000000e+00	3.268576	0.12286943
99	0.6399498	0.02556564	-0.36005016	0.5898412	0.6900585	0.000000e+00	3.932601	0.15029770
80	0.6839299	0.03500694	-0.31607013	0.6153163	0.7525435	0.000000e+00	4.928068	0.23544563
70	0.7062854	0.03399879	-0.29371461	0.6396478	0.7729230	0.000000e+00	5.259064	0.23750546
60	0.7298207	0.03209315	-0.27017933	0.6669181	0.7927232	0.000000e+00	5.575612	0.23176311
50	0.7557176	0.02961498	-0.24428238	0.6976723	0.8137630	2.220446e-16	5.899341	0.21988492
40	0.7855666	0.02697002	-0.21443342	0.7327053	0.8384278	1.776357e-15	6.249763	0.20268872
30	0.8220989	0.02531321	-0.17790112	0.7724850	0.8717128	2.095213e-12	6.654029	0.18257472
20	0.8712462	0.02819427	-0.12875384	0.8159854	0.9265069	4.955426e-06	7.165522	0.17260888
10	0.9517409	0.04511972	-0.04825907	0.8633063	1.0401756	2.848093e-01	7.943378	0.23568287
5	1.0300894	0.06966783	0.03008942	0.8935405	1.1666384	6.658154e-01	8.646160	0.36895879
2	1.1326403	0.10816823	0.13264027	0.9206305	1.3446500	2.201082e-01	9.503437	0.59274946
1	1.2103851	0.14078794	0.21038505	0.9344407	1.4863294	1.350868e-01	10.114634	0.78322652
0.5	1.2887785	0.17615178	0.28877851	0.9435210	1.6340360	1.011356e-01	10.702325	0.98752619
0.2	1.3939205	0.22701557	0.39392053	0.9489700	1.8388711	8.270296e-02	11.451461	1.27584661

$CAy

	combInd	seCI	CAdiff	ciloCAdiff	cihiCAdiff	CAdiffp	PredAdd	sePredAdd
-0.2	-6.246141	15.98104655	-7.2461407	-37.56899190	25.076711	6.502457e-01	1.274513	0.2445852
-0.5	-19.394622	120.00348198	-20.3946216	-254.60144624	215.812203	8.650493e-01	1.443815	0.2728977
-1	12.191095	33.44643940	11.1910951	-53.36392614	77.746116	7.379287e-01	1.725801	0.3687187
-2	3.991448	2.21941650	2.9914484	-0.35860794	8.341505	1.777055e-01	2.289397	0.5466498
-5	2.128313	0.50514537	1.1283131	1.13822818	3.118398	2.550674e-02	3.979126	0.8707750
-10	1.686106	0.28392387	0.6861065	1.12961568	2.242597	1.566985e-02	6.794470	1.0905217
-20	1.488006	0.14727617	0.4880064	1.19934505	1.776668	9.212100e-04	12.424572	1.1688759
-30	1.424987	0.10187579	0.4249871	1.22531058	1.624664	3.024410e-05	18.054504	1.2161818
-40	1.394013	0.08747537	0.3940133	1.22256162	1.565465	6.659910e-06	23.684387	1.3707492
-50	1.375597	0.08196254	0.3755970	1.21495038	1.536244	4.593528e-06	29.314251	1.5799862
-60	1.363389	0.07675733	0.3633885	1.21294418	1.513833	2.198644e-06	34.944103	1.7605632
-70	1.354702	0.06969076	0.3547019	1.21810797	1.491296	3.587174e-07	40.573950	1.8677796
-80	1.348205	0.06198961	0.3482051	1.22670549	1.469705	1.941297e-08	46.203793	1.9325552
-90	1.343163	0.05981961	0.3431629	1.22591650	1.460409	9.657907e-09	51.833633	2.1703342
-99	1.339502	0.07761812	0.3395018	1.18737028	1.491633	1.219894e-05	56.900487	3.1048805
99	1.339502	0.07750054	0.3395018	1.18760072	1.491403	1.183326e-05	56.900487	3.0886847
80	1.348205	0.06306100	0.3482051	1.22460557	1.471805	3.356992e-08	46.203793	2.0329821
70	1.354702	0.07300699	0.3547019	1.21160816	1.497796	1.182997e-06	40.573950	2.0610678
60	1.363389	0.08273892	0.3633885	1.20122026	1.525557	1.123176e-05	34.944103	2.0158471
50	1.375597	0.09101584	0.3755970	1.19720591	1.553988	3.679729e-05	29.314251	1.8648365
40	1.394013	0.09968792	0.3940133	1.19862501	1.589402	7.734919e-05	23.684387	1.6443250
30	1.424987	0.11575340	0.4249871	1.19811046	1.651864	2.411433e-04	18.054504	1.4222600
20	1.488006	0.15814184	0.4880064	1.17804835	1.797964	2.029524e-03	12.424572	1.2599316
10	1.686106	0.28267095	0.6861065	1.13207141	2.240142	1.521475e-02	6.794470	1.0819760
5	2.128313	0.47901587	1.1283131	1.18944201	3.067184	1.849871e-02	3.979126	0.8399322
2	3.991448	2.08412857	2.9914484	-0.09344358	8.076340	1.511881e-01	2.289397	0.5196061
1	12.191095	32.58457772	11.1910951	-51.67467725	76.056867	7.312618e-01	1.725801	0.3510270
0.5	-19.394622	118.62396439	-20.3946216	-251.89759175	213.108349	8.634952e-01	1.443815	0.2646745
0.2	-6.246141	15.91107245	-7.2461407	-37.43184268	24.939561	6.488107e-01	1.274513	0.2433053

#Plot the CI for the x axis:

```
plotFACI(0.355,list(ZnCd.lgau2, Zn.lgau2, Cd.lgau2), "x", ylim = c(-0.2, 2.2), faValues = c(-10, -20, -30, -40, -50, -60, -70, -80, -90, -99, 99, 90, 80, 70, 60, 50, 40, 30, 20, 10), showPoints = TRUE)

title("Combination index for x axis")
```

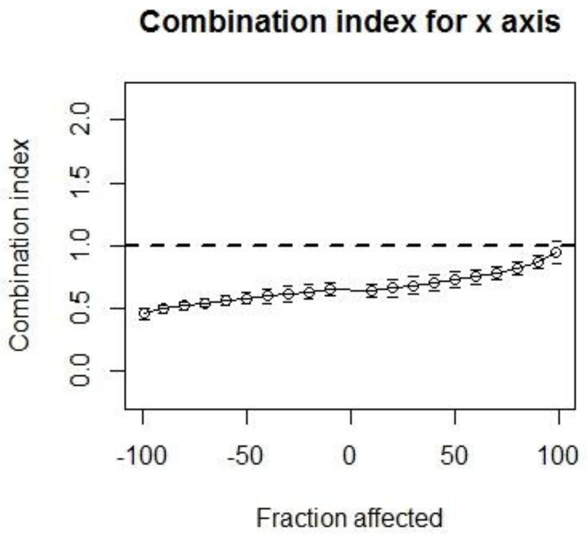

#Plot CI for the y axis

```
plotFACI(0.355,list(ZnCd.lgau2, Zn.lgau2, Cd.lgau2), "y", ylim = c(-0.2, 2.2), faValues = c(-10, -20, -30, -40, -50, -60, -70, -80, -90, -99, 99, 90, 80, 70, 60, 50, 40, 30, 20, 10), showPoints = TRUE)

title("Combination index for y axis")
```

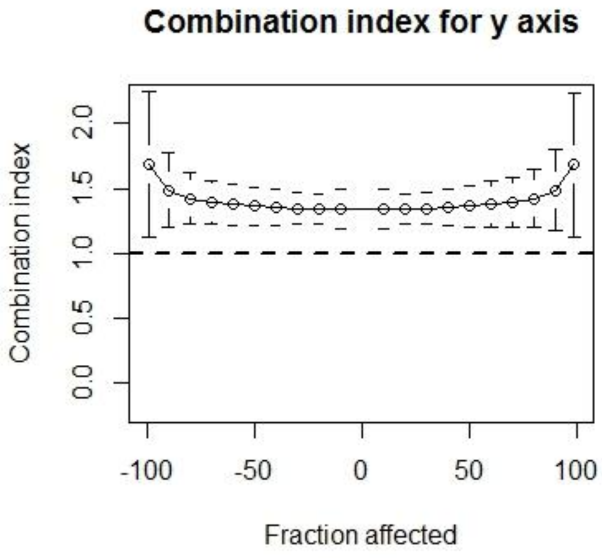

Supplementary Material SM4 (Figure).

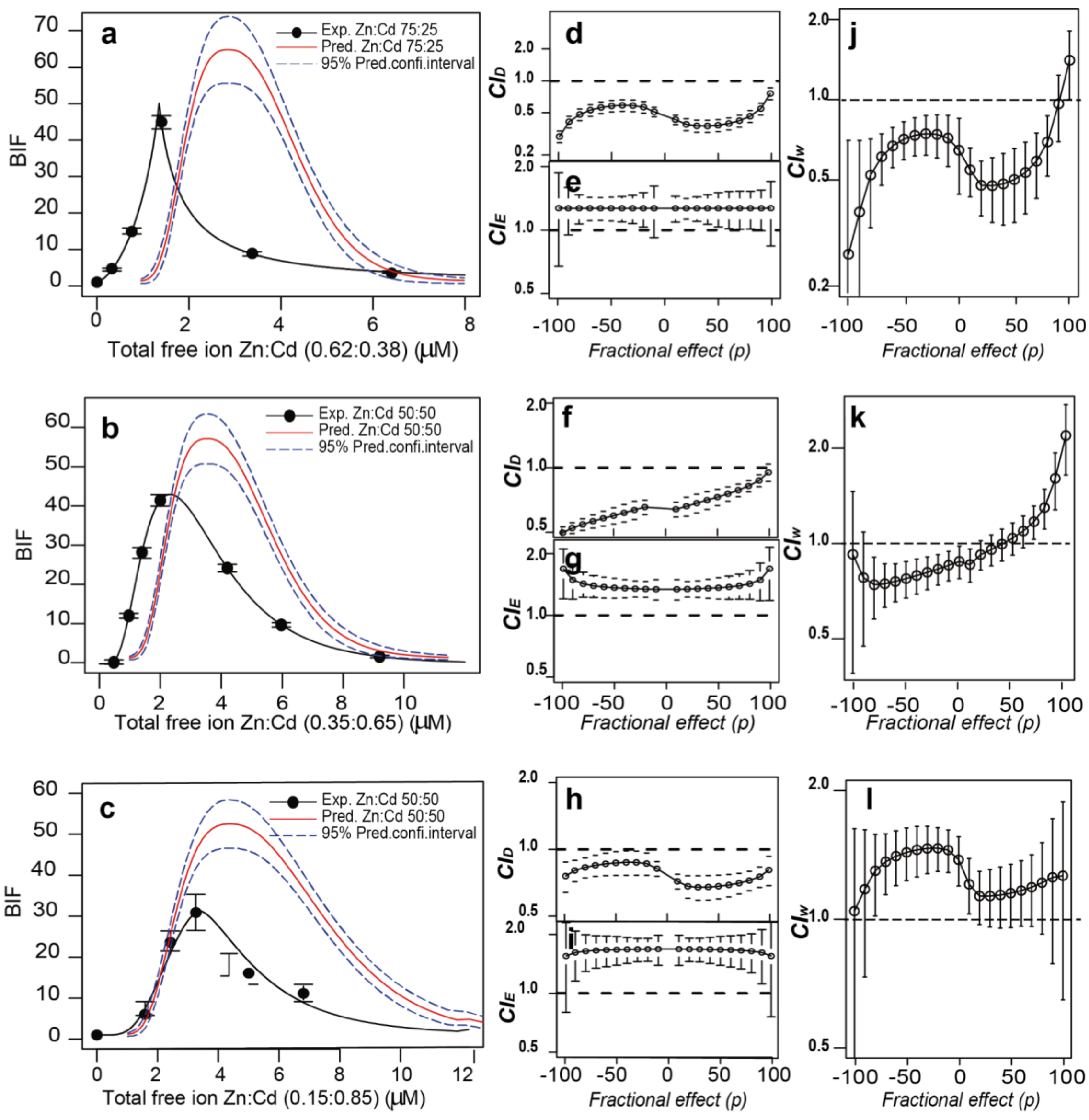

Effect of mixture ratio for the Zn:Cd binary mixture. Experimental *vs* predicted (under additivity) dose-response patterns for the mixture Zn:Cd at 75:25 (a), 50:50 (b) and 25:75 (c) mixture ratios based on the D_0 concentration of the metals. Extended *p-CI* plots presenting departures from additivity (as CI values) as a function of the effect level (p) for the D dimension (d, f, h), and the E dimension (e, g, i). Extended p-CI_w plots presenting weighted departures from additivity (as CI_w values) as a function of the effect level (p) for the binary mixture Zn:Cd at 75:25 (j), 50:50 (k), 25:75 (l) mixture ratios. Error bars are standard errors (n = 3-4). Total free ion concentrations presented in the Figures are those presented as ED_0 in Supplementary Material SM2 corrected by MINTEQ calculations due to the presence of the two metals used in each mixture.

Supplementary Material SM5 (Table). Combination index (CI) values for the dose (*D*) and empirical effect (*E*) dimensions together with relevant statistical information for 3 selected fractional effect levels (*p*) of the 15 binary metal mixtures of Cu, Cd, Zn, Cu, Hg and Co.

**Metal mixture**	Fractional affect (p)	CI_D	*p*-values	CI_E	*p*-values	CI*w*
Cu:Cd	-50	0.89± 0.05	0.03	1.48± 0.15	< 0.01	1.32± 0.2
	0	0.86± 0.03	< 0.01	1.52± 0.14	< 0.01	1.31±0.17
	50	0.77± 0.03	< 0.01	1.48± 0.18	< 0.01	1.14±0.21
Cu:Zn	-50	1.28± 0.05	< 0.01	0.71± 0.08	< 0.01	0.91±0.13
	0	1.11± 0.04	< 0.01	0.7± 0.08	< 0.01	0.77± 0.12
	50	1.07± 0.04	0.04	0.71±0.13	0.02	0.76±0.17
Zn:Cd	-50	0.56± 0.02	< 0.01	1.37± 0.08	< 0.01	0.77±0.1
	0	0.64± 0.02	< 0.01	1.34± 0.08	< 0.01	0.86±0.1
	50	0.75± 0.03	< 0.01	1.37± 0.09	< 0.01	1.03±0.12
Cu:Hg	-50	1.18±0.07	< 0.01	0.58±0.06	< 0.01	0.68±0.13
	0	1.89±0.07	< 0.01	0.59±0.03	< 0.01	1.11±0.10
	50	2.19±0.13	< 0.01	0.58±0.05	< 0.01	1.27±0.18
Co:Cd	-50	1.05 ± 0.03	0.01	1.03±0.09	0.76	1.08±0.12
	0	0.73± 0.02	< 0.01	1.04±0.06	0.45	0.76±0.08
	50	0.53± 0.02	< 0.01	1.03±0.06	0.68	0.54±0.08
Hg:Cd	-50	1.39±0.06	< 0.01	0.69±0.06	< 0.01	0.95±0.12
	0	1.22±0.02	< 0.01	0.69±0.04	< 0.01	0.84±0.06
	50	1.04±0.05	< 0.01	0.69±0.05	< 0.01	0.72±0.10
Ag:Cd	-50	1.41±0.04	< 0.01	0.64±0.04	< 0.01	0.9±0.08
	0	1.07±0.02	< 0.01	0.64±0.04	< 0.01	0.68±0.06
	50	0.73±0.02	< 0.01	0.64±0.04	< 0.01	0.47±0.06§
Zn:Hg	-50	3.61±0.23	< 0.01	0.52±0.05	< 0.01	1.87±0.28
	0	3.10±0.07	< 0.01	0.52±0.06	< 0.01	1.61±0.13
	50	2.45±0.16	< 0.01	0.52±0.09	< 0.01	1.27±0.25
Hg:Co	-50	1.11±0.06	< 0.01	0.29±0.09	< 0.01	0.32±0.15§
	0	1.24±0.04	< 0.01	0.28±0.02	< 0.01	0.35±0.06§
	50	1.17±0.07	< 0.01	0.29±0.02	< 0.01	0.34±0.09§
Cu:Ag	-50	1.13±0.03	< 0.01	0.68±0.04	< 0.01	0.77±0.07
	0	0.97±0.02	0.02	0.68±0.04	< 0.01	0.66±0.06
	50	1.06±0.02	< 0.01	0.68±0.09	< 0.01	0.72±0.11
Zn:Ag	-50	1.54±0.05	< 0.01	0.62±0.05	< 0.01	0.95±0.1
	0	1.07±0.04	< 0.01	0.61±0.06	< 0.01	0.65±0.1
	50	0.94±0.03	0.05	0.62±0.09	< 0.01	0.57±0.12
Hg:Ag	-50	1.26±0.06	< 0.01	1.62±0.10	< 0.01	2.04±0.16§
	0	1.32±0.03	< 0.01	1.58±0.09	< 0.01	2.08±0.12§
	50	1.26±0.05	< 0.01	1.62±0.25	0.01	2.04±0.3§
Co:Ag	-50	1.11±0.07	0.01	1.71±0.27	< 0.01	1.9±0.34
	0	1±0.05	0.94	1.87±0.18	< 0.01	1.87±0.23
	50	0.91±0.03	< 0.01	1.71±0.16	< 0.01	1.56±0.19
Zn:Co	-50	1.1±0.04	0.02	0.50±0.05	< 0.01	0.55±0.09
	0	0.85±0.04	< 0.01	0.49±0.03	< 0.01	0.42±0.07§
	50	0.7±0.03	< 0.01	0.5±0.04	< 0.01	0.35±0.07§
Cu:Co	-50	0.92±0.03	< 0.01	0.87±0.14	0.33	0.8±0.17
	0	1.85±0.04	< 0.01	0.88±0.06	0.04	1.63±0.1
	50	1.60±0.03	< 0.01	0.87±0.08	0.10	1.39±0.11

±: standard errors. For CI_D and CI_E, *P*.values were obtained using the function *Indices.fct* {drc}[1] which essentially performs a two-tailed Student's t-Test against the null hypothesis Ho: CI = 1. Statistical

significance is assumed to exist for *p*-values lesser than 0.05. For CI_w, it is not possible to directly perform a Student's t-Test since they are not directly computed with a dedicated *drc* function. In this case, statistical significance can be inferred from the absence of overlapping with 1 of the 95% confidence interval (IC95%= 1.95·Estandard deviation) for each CI_w value. However, in the paper and in the present Table, the criteria for the analysis of departures from additivity for CI_w is based on the pragmatic thresholds: Exp < 0.5 · CA (synergism) and Exp > 2 · CA (antagonism) [4,5], and it is marked by an §.

References

1 Ritz, C. & Streibig, J. C. Bioassay Analysis using R. *Journal of statistical softhware* **12**, 17 (2005).

2 The R Stats Package v. 3.3.0 (2015).

3 Martin-Betancor, K., Rodea-Palomares, I., Munoz-Martin, M. A., Leganes, F. & Fernandez-Pinas, F. Construction of a self-luminescent cyanobacterial bioreporter that detects a broad range of bioavailable heavy metals in aquatic environments. *Frontiers in microbiology* **6**, 186, doi:10.3389/fmicb.2015.00186 (2015).

4 Altenburger, R., Backhaus, T., Boedeker, W., Faust, M. & Scholze, M. Simplifying complexity: Mixture toxicity assessment in the last 20 years. *Environmental toxicology and chemistry / SETAC* **32**, 1685-1687, doi:10.1002/etc.2294 (2013).

5 Cedergreen, N. Quantifying Synergy: A Systematic Review of Mixture Toxicity Studies within Environmental Toxicology. *PloS one* **9**, e96580, doi:10.1371/journal.pone.0096580 (2014).
